# Constitutive interferon epsilon expression shapes antiviral epithelial states in the female reproductive tract and intestine

**DOI:** 10.1128/mbio.00340-26

**Published:** 2026-05-05

**Authors:** Rebecca L. Casazza, Samantha Skavicus, David Hare, Kaila A. Cooley, Nicholas S. Heaton, Carolyn B. Coyne

**Affiliations:** 1 Department of Integrative Immunobiology, Duke University School of Medicine 12277 , Durham, North Carolina, USA; 2 Department of Microbiology and Molecular Genetics, Duke University School of Medicine 12277 , Durham, North Carolina, USA; 3 Duke Human Vaccine Institute 188743, Durham, North Carolina, USA; Tsinghua University, Beijing, China

**Keywords:** interferons, mucosal immunity, enteric viruses, reproductive immunology, gastrointestinal infection

## Abstract

Antiviral defenses at mucosal barriers are essential for preventing viral entry and systemic infection. Interferon epsilon (IFNε) is a unique type I IFN that, unlike other family members, is not induced by infection but is constitutively expressed in epithelial tissues. IFNε was initially characterized in the female reproductive tract (FRT), where it provides broad antiviral protection, but its roles outside the FRT remain poorly defined. Here, we used *Ifnε^−/−^* mice and single-cell RNA sequencing to delineate IFNε function across distinct mucosal surfaces. In the FRT, *Ifnε* expression was restricted to specific epithelial subsets, was independent of estrous stage, and maintained basal ISG expression. IFNε was also retained intracellularly in primary human FRT-derived cells. Extending these analyses to the intestine, we found that IFNε is highly expressed in villous-tip enterocytes of the small intestine *in vivo*, where it sustains inflammatory enterocyte subsets and maintains type III IFN expression. Loss of *Ifnε* depleted these subsets and rendered mice more susceptible to enteric viral infection. Together, these findings establish IFNε as a constitutively expressed, spatially restricted IFN that coordinates mucosal antiviral defenses across both reproductive and gastrointestinal epithelial tissues.

**IMPORTANCE:**

Interferon epsilon (IFNε) is a unique type I IFN that, unlike other family members, is not induced by infection but is constitutively expressed in epithelial tissues. In this manuscript, we define the epithelial cell types that constitutively express IFNε in the uterus and small intestine at a single-cell resolution. We show that mice lacking IFNε lose key antiviral defenses in a tissue-dependent manner; uterine epithelial cells have diminished basal ISG expression, and key populations of cytokine-expressing enterocytes are absent from the small intestine. In the intestine, this correlates with increased susceptibility to infection with an enteric virus in mice. These findings establish IFNε as a key contributor to mucosal immunity, sustaining antiviral defenses within tissue-specific epithelial cells of both the female reproductive tract and intestine, and broaden our understanding of its role beyond traditional pathogen-induced interferon responses.

## INTRODUCTION

Antiviral defenses at barrier sites are essential for preventing entry and dissemination of viruses, which could compromise tissue integrity and lead to systemic infection. In the reproductive tract, these defenses are particularly crucial, given the exposure to external pathogens, such as sexually transmitted viruses, and their essential role in safeguarding reproductive health and ensuring successful reproduction. Similarly, in the intestine, antiviral programs are required to control constant exposure to enteric viruses while maintaining epithelial integrity and nutrient absorption. In both tissues, robust antiviral responses are needed to preserve a balanced immune environment, minimizing the risk of infection while sustaining barrier function and overall tissue homeostasis.

Interferon epsilon (IFNε) is a type I IFN that has been described as specifically enriched in the epithelial cells of the female reproductive tract (FRT) (1, 2). Unlike other type I IFNs, IFNε is constitutively expressed and is not induced by viral infections. Despite its lack of induction by infection, IFNε plays a direct role in innate antimicrobial defense against sexually transmitted infections, including HIV, herpes simplex virus (HSV), Zika virus (ZIKV), and chlamydia (1, 3, 4). Beyond antiviral defense, IFNε exerts tumor-suppressive activity in fallopian tube epithelial cells (5) and has been implicated in pregnancy, as it is expressed in the myometrium, cervix, and chorioamniotic membranes (6). Elevated levels of IFNε in amniotic fluid during spontaneous preterm labor with intra-amniotic infection suggest a role in antimicrobial defense within the amniotic cavity. However, consistent with viral infections, IFNε expression is not enhanced by bacterial stimulation *in vitro*, indicating that alternative cellular sources may contribute to its presence during infection (1, 6).

Several clues exist as to the regulation of *IFNε* expression. Analysis of the *IFNε* promoter region revealed progesterone response elements implicating hormonal modulation in its expression (7). Consistent with this, *Ifnε* expression fluctuates in the mouse uterus with the estrous cycle and peaks during estrous when progesterone levels are the highest (1). The transcription factor ELF3, which directly regulates the expression of genes important for maintaining the integrity and function of epithelial barriers, contributes to the control of IFNε expression in the uterus, and the levels fluctuate with estrous status (8). Transcription factor binding site analysis has also revealed potential regulatory sites for NF-κB, a critical mediator in immune and inflammatory responses (7). NF-κB binding elements in the IFNε promoter suggest that this cytokine might be responsive not only to hormonal signals but also to cellular stress and inflammatory stimuli, which aligns with its role in epithelial immune defense. Indeed, treatment of cells with tumor necrosis factor-α (TNF-α) upregulates *IFNε* expression (9). Taken together, these findings suggest that *IFNε* expression may be finely tuned by both hormonal and inflammatory cues, with transcription factors including NF-κB and ELF3 potentially coordinating epithelial immune readiness in response to the estrous phase and/or local environmental stimuli.

In addition to antimicrobial and antitumor properties in the epithelium of the FRT, IFNε potentially plays a role in modulating the immune system. For example, *Ifnε^−/−^* mice exhibit less uterine NK cell accumulation at baseline and less activation after infection than WT mice, a phenotype which results in part from differences in cytokine secretion in *Ifnε^−/−^* macrophages/monocytes (10). Additionally, T, B, and NK cells respond to recombinant IFN*ε in vitro* (4). These findings suggest that IFNε not only fortifies epithelial defenses but also plays a broader immunoregulatory role in the FRT, coordinating both innate and adaptive immune responses to maintain tissue homeostasis and readiness against infection.

Recent studies have suggested that IFNε might also play a role in inflammatory signaling outside of the FRT. In addition to epithelial cells of the FRT, *Ifnε* is expressed in the intestinal epithelium, where it helps limit inflammation and supports the intestinal regulatory T cell compartment (11). In the testes, Ifnε is expressed in the germinal epithelium in spermatogenic cells, as well as in Leydig cells and macrophages, where it protects against infection (12). Ifnε is also constitutively expressed in human primary bronchial epithelial cells and in the intestines of bats (13–15). In rhesus macaques, IFNε is expressed in the epithelium of the large and small intestines, lung, and foreskin in addition to the female reproductive tract (16). These studies highlight IFNε as a multifaceted cytokine with roles in maintaining immune balance across diverse mucosal tissues, suggesting that it may provide a widespread defense mechanism beyond the FRT.

The mechanisms regulating IFNε and its contributions to antiviral defense beyond the female reproductive tract (FRT) remain incompletely defined. To address this, we generated an IFNε knockout (*Ifnε*^−/−^) mouse using a modified intracytoplasmic gene editing via oviductal nucleic acids delivery (iGONAD) approach, which enables *in vivo* genetic modification without embryo transfer (17, 18). Consistent with a previously described *Ifnε*^−/−^ model (1), we confirmed that IFNε protects against intravaginal herpes simplex virus-2 (HSV-2) infection. Analysis of single-cell RNA sequencing (scRNA-Seq) data sets across the estrous cycle in the mouse FRT (19) revealed that *Ifnε* is expressed in tissue-specific epithelial cell clusters and is not directly regulated by the estrous cycle. Instead, expression varies with fluctuations in the abundance of Ifnε-expressing epithelial subsets. These populations exhibited high interferon-stimulated gene (ISG) expression, which was absent in epithelial subsets lacking *Ifnε*, suggesting an autocrine mechanism of action. Supporting this, scRNA-seq of uterine tissues from WT and *Ifnε*^−/−^ mice showed significant reductions in ISG expression specifically within Ifnε-expressing epithelial subsets, without broader transcriptional effects. In primary human FRT epithelial cells, IFNε was constitutively expressed, non-inducible by pathogens, and retained intracellularly. Similarly, IFNε was constitutively expressed and retained intracellularly in human stem cell-derived enteroids. Single-cell RNA sequencing of human enteroids and murine small intestine identified IFNε expression specifically in mature villous-tip enterocytes, establishing that IFNε is constitutively expressed within this epithelial subset *in vivo*. Mice lacking *Ifnε* exhibited reduced expression of antimicrobial genes, loss of inflammatory enterocyte populations that normally produce cytokines such as Ifnλ3, and increased susceptibility to oral infection with the enterovirus coxsackievirus B (CVB). These findings establish IFNε as a key contributor to mucosal immunity, sustaining antiviral defenses within tissue-specific epithelial cells of both the FRT and intestine, and broaden our understanding of its role beyond traditional pathogen-induced interferon responses.

## RESULTS

### Generation of *Ifnε^−/−^* mice using iGONAD

**Fig 1:**
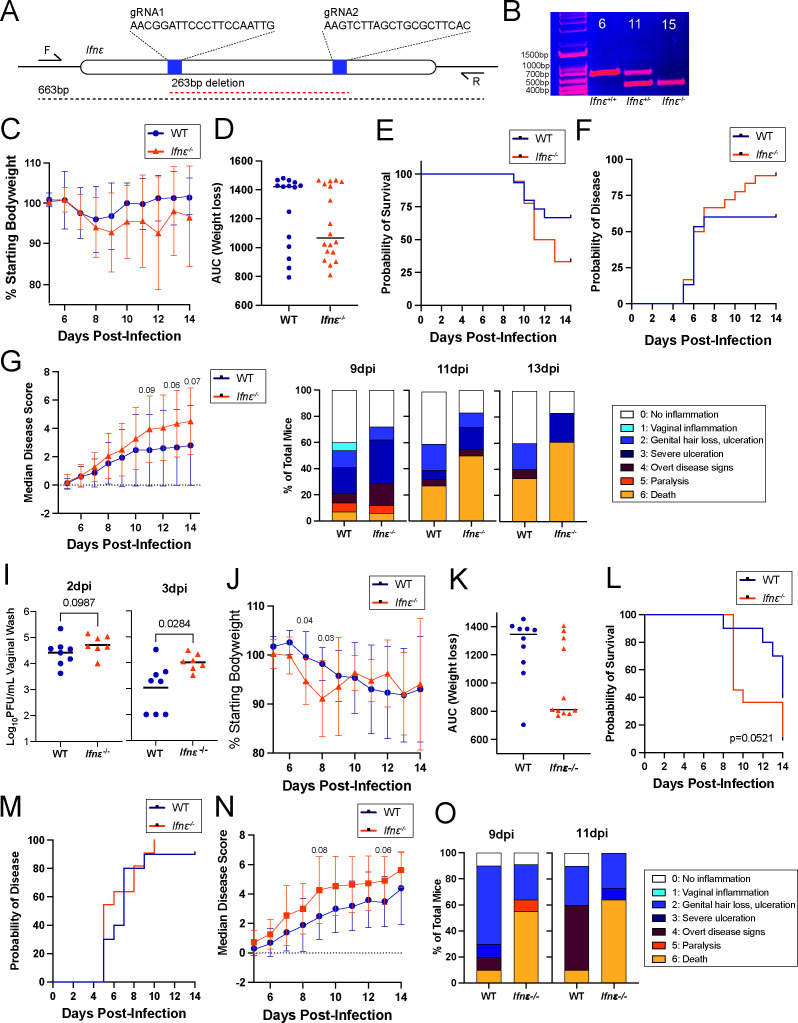
Increased HSV-2 susceptibility in *Ifnε*-deficient mice generated via iGONAD CRISPR/Cas9. (**A**) Schematic of CRISPR/Cas9 knockout strategy. (**B**) Representative PCR genotyping results from the first generation of pups born to dams that underwent iGONAD gene editing. Mice 6 (*Ifnε^+/+^),* 11 (*Ifnε^+/−^*), and 15 (*Ifnε^−/−^*) are shown. Mouse 15 was chosen as the founding sire for the line. (**C–H**) WT (*n* = 15) and *Ifnε^−/−^* (*n* = 18) females were infected with 100 PFU of HSV-2 333 and weighed and monitored for disease signs daily. Weight loss (**C and D**), survival (**E**), and disease onset (**F**) are shown. Disease onset (**F**) includes any observable pathology score. Median clinical score is shown in panel **G**, and in panel **H**, frequencies of clinical scores are shown at multiple days post-infection (dpi) from 9 to 13 dpi. Key at right. Differences in median clinical score were determined by the Mann-Whitney *U* test in Graphpad Prism. (**I and J**) WT (**I** *n* = 8, **J–O** *n* = 10) and *Ifnε^−/−^* (**I** *n* = 7, **J–O** *n* = 11) females were infected with 10,000 PFU of HSV-2 333 and lavaged vaginally or monitored for weight loss, survival, and disease signs daily. Vaginal lavages were collected at 2 and 3 dpi and titrated by plaque assay. Significant differences were calculated by *t*-test in GraphPad Prism. Individual animals are depicted by symbols in panel **I**. Weight loss (**J and K**), survival (**L**), and disease onset (**M**) are shown. Median clinical score is shown (**N**), and frequencies of clinical scores are shown at peak disease 9–11 dpi in (**J**). Significant differences in weight loss and median disease score were calculated by the Mann-Whitney U test in GraphPad Prism. Differences in survival outcomes were calculated by χ^2^ in GraphPad Prism.

We used a modified iGONAD protocol (18) to generate an *Ifnε^−/−^* knockout mouse. The iGONAD technique was chosen for its efficiency and precision in gene editing, particularly in the context of creating knockout models. To achieve this, we employed a dual sgRNA approach targeting the single coding exon of the *Ifnε* gene, which resulted in a 263 base pair deletion (Fig. 1A). Fifteen total pups were born following the injection and electroporation of sgRNAs and Cas9 into the oviducts of pregnant dams. These pups were subsequently screened for *Ifnε* knockout by PCR using primers spanning both gRNA target sites. The genotyping results revealed that 6 out of the 15 pups were homozygous for the *Ifnε* deletion, indicating a successful knockout of both alleles. Additionally, three pups were identified as heterozygous, carrying one wild-type and one knockout allele, while the remaining six pups were wild type (representative genotypes, Fig. 1B). Successful gene knockout was further validated by Sanger sequencing, which confirmed the deletion at the nucleotide level (Fig. S1A). Following this, a qPCR-based genotyping assay was developed in collaboration with a commercial vendor (Transnetyx) to streamline the genotyping process for subsequent generations. To establish a stable knockout line, one animal (15) identified as homozygous for the *Ifnε* deletion was selected as the founding sire. To ensure genetic integrity and minimize potential off-target effects, this animal was backcrossed ~six times with C57Bl/6J mice. Following backcrossing, a stable and reproducible *Ifnε*^−/−^ line was developed and used for subsequent studies.

To confirm that this newly established *Ifnε^−/−^* line recapitulated phenotypes observed in a previous *in vivo* model (1) regarding the role of IFNε in antiviral signaling in the FRT, we infected *Ifnε^−/−^* and WT female mice intravaginally with 100 PFU of HSV-2 (strain 333) and scored mice daily for clinical signs. *Ifnε^−/−^* mice lost more weight compared to their WT counterparts (Fig. 1C and D), a higher percentage of *Ifnε^−/−^* mice succumbed to infection (73% vs. 40%) (Fig. 1E), a higher percentage of *Ifnε^−/−^* mice had disease onset compared to WT mice (89% vs. 60%), defined as any observable clinical sign (Fig. 1F), and *Ifnε^−/−^* mice displayed higher frequencies of advanced clinical signs throughout the course of infection (Fig. 1G and H). Despite *Ifnε^−/−^* having worse disease outcomes by every metric measured, none of these differences were significant, in part, due to a large proportion of mice that remained uninfected. To increase the infection rates in our mice, we performed infections with 10,000 PFU of HSV-2 (Fig. 1I through O). *Ifnε^−/−^* mice had higher viral burdens in vaginal lavages at 2 and 3 dpi (Fig. 1I) and lost significantly more weight before succumbing to infection (Fig. 1J and K). A higher percentage of *Ifnε^−/−^* mice succumbed to infection (91% vs. 60%, *P* = 0.0521, as measured by χ² test) despite having similar rates of disease onset (Fig. 1L and M). *Ifnε^−/−^* mice had worse clinical outcomes measured by clinical scores throughout infection (median of 2 in WT mice vs. median of 6 in *Ifnε^−/−^* mice at peak disease [9 dpi], *P* = 0.0751, as measured by Mann-Whitney *U* test) (Fig. 1N and O). Taken together, these data show that *Ifnε^−/−^* mice generated using iGONAD recapitulate the heightened susceptibility and impaired antiviral response observed in a previous *in vivo* model of HSV-2 infection in the FRT.

### IFNε is a key modulator of basal ISG expression in the FRT epithelium

**Fig 2:**
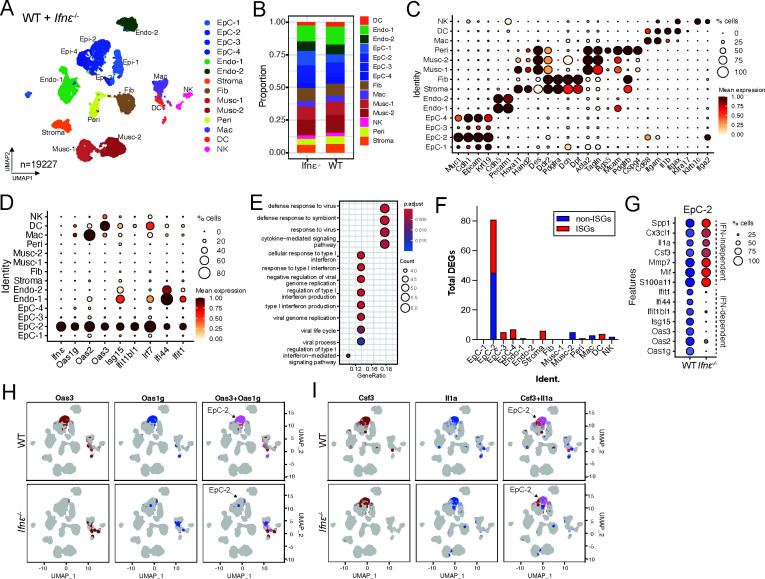
*Ifn*ε^–/–^ mice lack basal ISG expression in uterine epithelial cells. (**A**) UMAPs showing uterine cell clusters from *Ifn*ε*^−/−^* and WT mice. (**B**) Bar plot of the proportions of each cell cluster in *Ifnε^−/−^* and WT mice. (**C**) Dot plot of canonical cell type markers in each cell cluster. Key and scale are shown on the right side. (**D**) Dot plot of *Ifnε* and ISG expression in each cell cluster. Key and scale are shown on the right side. (**E**) Pathway analysis on differentially expressed genes (DEGs) in *Ifnε^−/−^* and WT uteruses. Gene counts per pathway and the *P*-value of each pathway are shown. (**F**) The total number of DEGs in each cluster. ISGs are shown in red, and non-ISGs are shown in blue. (**G**) Dot plot shows ISG and cytokine expression levels split by mouse genotype. Scale is shown on the right side. (**H**) Feature plots show the co-expression of *Oas3* and *Oas1g* in either WT (top) or *Ifnε^−/−^* (bottom) samples. (**I**) Feature plots show the co-expression of *Csf3* and *Il1a* in either WT or *Ifnε^−/−^* samples.

To determine the specific cell types expressing *Ifnε* in the uterus and the functional consequences of *Ifnε* signaling, we performed scRNA-seq on uterine tissues isolated from WT and *Ifnε^−/−^* mice in estrous, with 19,227 cells passing quality control. Cluster analysis and cell type-specific marker expression following integration to correct for batch effects across independent animals revealed four distinct clusters of EpCs, two populations of endothelial cells (Endo), stroma and fibroblasts, two populations of muscle cells (Musc-1 and Musc-2), pericytes (Peri), and various immune cells including macrophages (Mac), dendritic cells (DC), and NK cells (NK) (Fig. 2A). There were near-equivalent ratios of all cell types between WT and *Ifnε^−/−^* mice (Fig. 2B; Fig. S2A) and cell clusters expressed cell type-specific markers, which were conserved between WT and *Ifnε^−/−^* mice (Fig. 2C; Fig. S2B). We identified a single cluster of EpCs (EpC-2) that highly expressed *Ifnε* and that also expressed high basal ISG levels (Fig. 2D). This cluster also expressed markers associated with luminal epithelial cells (LEpCs) (e.g., *Prap1*, *Tacstd2*, and *Ltf*) (Fig. S2C and D). Luminal epithelial cells line the inside of the uterus, expand and contract with the estrous cycle, and would be the cell layer that an embryo would implant into.

To assess whether *Ifnε* expression was required for basal ISG expression in EpC-2 and other cell types, we performed pseudobulk differential expression analysis across all cell clusters in WT and *Ifnε*^−/−^ mice. We identified a total of 115 differentially downregulated transcripts in *Ifnε^−/−^* mice compared to WT, with ~70% of these DEGs attributed specifically to the EpC-2 cluster (Fig. S2E). Pathway analysis of these DEGs showed a significant enrichment in genes associated with IFN signaling and the antiviral response (Fig. 2E). Of the 81 differentially downregulated DEGs in EpC-2, ~45% of them were ISGs (Fig. 2F). Several additional clusters also exhibited a downregulation of select ISGs (EpC-3, EpC-4, Stroma, and DC), but the total number of DEGs was significantly lower than that observed in EpC-2 (Fig. 2F). Mac and DC clusters, which also express basal ISGs, had minimal DEGs. (Fig. 2F). There was no differential expression of ISGs in the Mac cluster, indicating that the basal ISG expression in this cluster is Ifnε-independent. However, in DCs, there were four ISGs significantly downregulated in *Ifnε^−/−^* samples (Rsad2, Ifi213, Ifi44, and Isg15). Although this does not account for all the ISGs basally expressed in the DC cluster, it may indicate a role for Ifnε in modulating ISG expression in dendritic cells. Additional clusters, including EpC-1, Endo-2, Fib, and Musc-1, did not exhibit any changes in gene expression in *Ifnε^−/−^* mice (Fig. 2F). There were only two DEGs enriched in *Ifnε^−/−^* mice, *Ppbp* and *Crct*. Importantly, although ISGs were significantly downregulated in EpC-2, other non-IFN-mediated inflammatory transcripts were unchanged, including those associated with IL1 signaling (e.g., *Il1a*), chemokine signaling (e.g., *Cx3cl1*), and cytokine signaling (e.g., *Mif*) (Fig. 2G through I). Taken together, these data show that *Ifnε* expression is crucial for maintaining basal ISG expression in EpC populations in the FRT.

### *Ifnε* is expressed in epithelial cells of the oviduct, uterus, cervix, and vagina, independent of estrous status

**Fig 3:**
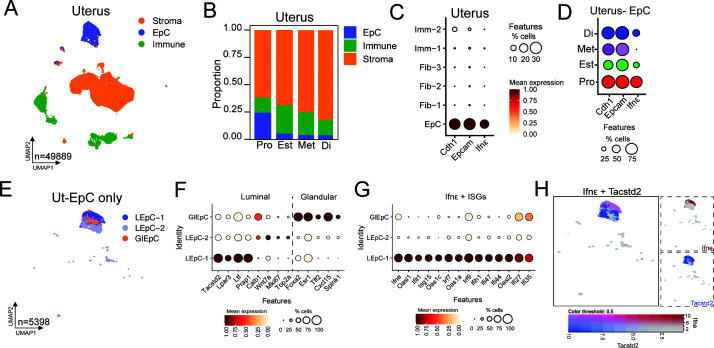
*Ifnε* is expressed in epithelial cells of the female reproductive tract throughout the estrous cycle. (**A**) UMAP showing the clustering of stromal (Stroma, orange), epithelial (EpC, blue), immune (Immune, green), endothelial (EndoC, red), and muscular (Musc, yellow) cells within the different tissues of the FRT. (**B**) The proportions of each cell type split by estrous stage: proestrous (Pro), wstrous (Est), metestrous (Met), and diestrous (Di). (**C and D**) Dot plots show the expression of epithelial cell markers and *Ifnε* in all clusters (**C**) or only in clusters that express *Ifnε,* split by estrous stage (**D**). (**E–H**) EpC cells were subsetted and reclustered into glandular epithelial cell (GlEpc) and luminal epithelial cells (LEpC). (**E**) UMAP showing clustering of distinct epithelial populations. (**F–G**) Dot plots showing expression of key glandular and luminar markers used to identify clusters (**F**) or expression of *Ifne* and ISGs (**G**). (**H**) A feature plot showing the overlap of the luminal epithelial marker, *Tacstd2*, and *Ifne* expression within epithelial cells.

To determine the impact of the estrous cycle on the expression of *Ifnε* in the FRT, we analyzed an existing single-cell atlas of the cycling mouse female reproductive tract based on 378,516 cells from normal cycling young mice in the four cycle phases (proestrus [Pro], estrus [Est], metestrus [Met], and diestrus [Di]) (19). The data set contained cells harvested from the ovary, oviduct, uterus, cervix, and vagina from 3 to 5 independent animals. We restricted our analyses to non-pregnant young mice (aged <12 months). A total of 71,173 (ovary), 75,091 (oviduct), 49,889 (uterus), 78,967 (cervix), and 82,861 (vagina) cells passed quality control and were used for cluster analysis and cell type-specific marker expression analysis following integration to correct for batch effects (Fig. 3A; Fig. S3A through J). We broadly clustered cells into epithelial cells (EpC), stromal cells, immune cells, endothelial cells (EndoC), or muscle cells. Each tissue site exhibited site-specific enrichment of these cell types, consistent with what has been described (19), with the cervix and vagina most enriched in EpCs, and the ovary, oviduct, and uterus more enriched in stromal cells (Fig. 3A; Fig. S3A through J). While there was a slight variation in the proportion of these cell types during the estrous cycle, including the enrichment of immune cell types in the cervix and vagina during Met, the proportion of cell types was largely maintained across all tissues (Fig. 3; Fig. S3A through J).

We next determined the cell type(s) expressing *Ifnε* across all tissue sites and defined the impact of the estrous cycle on this expression. For this analysis, we further clustered cell types to increase resolution. In the oviduct, uterus, cervix, and vagina, we found that *Ifnε* was specifically enriched in EpC cell types that also expressed high levels of epithelial markers (e.g., *Cdh1* and *EpCam*) (Fig. 3C; Fig. S3L). We did not detect any *Ifnε* in the ovaries (Fig. S3L). We next determined the levels of *Ifnε* expressed in EpCs across the estrous cycle in all tissues. In the oviduct, cervix, and vagina, we observed highly similar levels of expression across all stages of the estrous cycle (Fig. S3M). In the uterus, levels of *Ifnε* were consistent across Pro, Est, and Di, with significantly lower levels in Met (Fig. 3D). Similarly, *Ifnε* levels were low in the uterus of pregnant mice (Fig. S3K and N). Taken together, these data indicate that IFNε is predominantly expressed in epithelial cell types across the FRT, with consistent expression levels across the estrous cycle, except in the uterus, where levels significantly decrease during Met and pregnancy.

### *Ifnε*-expressing epithelial cells in the FRT express high basal ISGs

The FRT is lined by specialized epithelial cell types, each with distinct functions tailored to their anatomical location. These include ciliated and secretory EpCs in the oviduct, luminal and glandular EpCs in the uterus, mucus-secreting EpCs in the cervix, and stratified squamous EpCs in the vagina. Given that *Ifnε* was expressed in EpC populations across the FRT, we next sought to identify the specific EpC populations responsible for this expression and whether it varied by location. Furthermore, we sought to investigate whether *Ifnε*-expressing epithelial cell populations exhibited distinct transcriptional profiles, including elevated ISG levels. To do this, we subsetted the EpC cell cluster from all anatomical sites and re-clustered to obtain better resolution between distinct EpC types. In the oviduct, EpC cells were clustered into ciliated (cEpC) and secretory (SEpC) populations, which were distinguished based upon marker expression (Fig. S4A and B). We found that *Ifnε* was specifically expressed in an SEpC cell population marked by *Serpina1e* expression (Fig. S4C and D). This population also exhibited high basal levels of ISGs (Fig. S4C). In the uterus, EpCs were distinguished between markers associated with luminal EpCs (LEpCs) and glandular EpCs (GlEpCs) (Fig. 3E and F), with *Ifnε* expression restricted to a LEpC population expressing high levels of Tactstd2 (Fig. 3F through H). This cluster also expressed high levels of diverse ISGs (Fig. 3G). The cervix contained the largest number of EpCs, which sub-clustered into intermediate (IEpCs), superficial (SuEpCs), and basal EpCs (BEpC), some of which were proliferating (pBEpC) (Fig. S4E and F). *Ifnε* was expressed in both SuEpC populations, with significantly higher levels in the Sprr2d^+^ cluster, which also showed elevated ISG expression (Fig. S4G and H). The vaginal EpCs clustered into SuEpCs, BEpCs, and columnar EpCs (ColEpCs), with ColEpCs expressing both *Ifnε* and basal ISGs (Fig. S4I through L). These data demonstrate that *Ifnε* is expressed in specific epithelial cell populations across tissue sites, with its expression correlating with high basal levels of ISG expression within these clusters.

The data described above suggested that *Ifnε* is expressed in distinct EpC types in an anatomical location-specific manner, suggesting that these cell types might exhibit conserved expression of factors responsible for *Ifnε* expression. To determine if this was the case, we compared the differentially enriched genes in all *Ifnε*-expressing clusters across anatomical sites and identified the genes shared between these cell types. This analysis revealed that only 31 genes (1% of the total genes) were shared between *Ifnε*-expressing EpC types (Fig. S5A). The top-most gene shared across all cell clusters was *Elf3* (Fig. S5B), which has been shown previously to regulate *Ifnε* expression (8). In fact, there were only two transcription factors shared between all *Ifnε*-expressing EpC clusters, which included *Elf3* and *Maff* (Fig. S5C). These findings suggest that despite the anatomical specificity of *Ifnε* expression, only a small set of conserved transcription factors, which included *Elf3*, may play a role in regulating *Ifnε* expression across diverse epithelial cell types.

### IFNε is retained intracellularly and is not induced by viral infections in primary human epithelial cells

**Fig 4:**
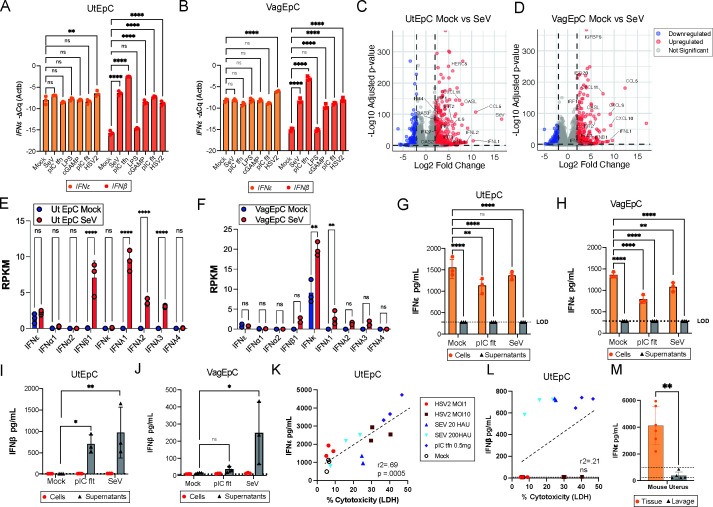
IFNε is retained intracellularly in primary human epithelial cells and is not induced by viral infections. (**A and B**) Primary human uterine (UtEpV) (**A**) or vaginal (VagEpC) (**B**) epithelial cells were infected with Sendai virus cantell (SeV, 200 HAU) or HSV2 (MOI of 1), treated with LPS or poly(I:C), or transfected with cGAMP or poly(I:C). Cells were harvested 20 hpi, and *IFN*ε and *IFN*β levels were quantified by qRT-PCR. (**C and D**) Volcano plots of differentially expressed genes (log_2_ ≥ 2 or ≤ −2, *P*_adj_ ≤0.01) in UtEpC (left) or VagEpC (right) infected for 20 h with 200 HAU SeV. Upregulated genes are shown in red, and downregulated genes are shown in blue. Select gene names are shown in black. (**E and F**) Reads per kilobase of transcript, per million mapped reads (RPKM) of the indicated IFN gene in mock-infected (blue) or SeV-infected (red) UtEpC (**D**) or VagEpC (**E**). (**G–J**), IFNε (**G and H**) and IFNβ (**I and J**) protein levels were measured from cell lysates and cell supernatants from uterine (**G and I**) or vaginal (**H and J**) epithelial cells by ELISA. Significance was determined by two-way ANOVA using a Tukey’s test for multiple comparisons (*, *P* < 0.05, **P < 0.01, ***P < 0.001, ****P < 0.0001; ns, not significant). (**K and L**) UtEpC were infected for 24 h with HSV-2 (MOI of 1 and 10) and SeV (20 and 200 HAU), or transfected with 0.5 mg of poly(I:C). Cellular supernatants were collected at 24 h, and IFNε (K) and IFNβ (**L**) were measured by ELISA. Cellular cytotoxicity was quantified by measuring lactate dehydrogenase activity and correlated to IFN levels by linear regression and Spearman’s correlation. (**M**) Eight-week-old female WT and Ifnε*^−/−^* mice were synchronized into estrous by depo-estradiol injection. Uteruses were harvested and lavaged with PBS. Ifnε levels were measured by ELISA in tissue lysate and lavage and compared by the Mann-Whitney *U* test in GraphPad Prism (*P* = 0.0032).

To assess whether IFNε expression levels changed following infection, we cultured human primary endometrial and vaginal epithelial cells, infected them with Sendai virus (SeV) or HSV-2, or treated them with a variety of PAMPs, including poly(I:C) treatment and transfection, cGAMP transfection, and LPS treatment, and measured *IFN*ε expression after a 20-h incubation time by qRT-PCR. Consistent with what we observed in the mouse scRNA-seq analysis described above, *IFNε* was basally expressed in both uterine and vaginal primary cells, and expression levels remained unchanged with infection or PAMP treatment (Fig. 4A and B). In contrast, *IFNβ* was undetected at baseline and was induced by all conditions except for LPS treatment (Fig. 4A and B). To broadly profile the induction of *IFNε* and other IFNs, we performed whole transcriptome bulk RNA-seq on uterine and vaginal epithelial cells infected with SeV, as well as on mock-infected controls. Both cell types responded to infection by induction of various IFNs, including *IFNβ* and *IFN*λ 1-3, as well as canonical ISGs (e.g., *OAS1*, *OASL*, and *ISG15*) (Fig. 4C through F). In contrast, although *IFNε* was expressed basally, it was not induced by SeV infection in either cell type (Fig. 4E and F).

In our scRNA-seq data sets, we observed basal ISG expression in clusters that also expressed *Ifnε*, suggesting an autocrine role for IFNε signaling in subsets of epithelial cells of the FRT. It has previously been observed *in vitro* that IFNε is not secreted efficiently and accumulates in the cytoplasm and endoplasmic reticulum rather than moving through the Golgi secretory pathway like other type I IFNs (20). To determine if IFNε is retained intracellularly in primary cells, we examined both lysates and supernatants from human uterine and vaginal primary epithelial cells for IFNε or IFNβ levels by ELISA. IFNε was undetectable in the supernatants from vaginal and uterine primary cells basally, after poly(I:C) treatment, and after SeV infection (Fig. 4G and H). However, there were high levels of IFNε detected in the cell lysates in all conditions, although there was significantly more IFNε at baseline than there was following poly(I:C) treatment or infection (Fig. 4G and H). In contrast, IFNβ was undetectable at baseline, and although it could be observed at low levels in cell lysates following poly(I:C) treatment or SeV infection, it was significantly higher in the supernatants (Fig. 4I and J).

We hypothesized that the high, basal levels of IFNε could function as a DAMP in the context of lytic infection or cellular damage. To test this, we examined IFN levels and lactate dehydrogenase (LDH) activity as an indicator of cytotoxicity in uterine epithelial primary cells supernatants 24 h following lytic infections (HSV-2, SeV) or poly(I:C) transfection. After 24 h, we were able to measure IFNε in the supernatant in all conditions tested, including 20 HAUs of SeV infection, which produced no detectable IFNε at our earlier 20-h time point (Fig. 4K). We found that IFNε concentrations correlated positively (linear fit *r*^2^ = 0.69, Spearman’s correlation *P* = 0.0005) with % cellular cytotoxicity measured by lactate dehydrogenase activity in the supernatant (Fig. 4K). In comparison, IFNβ levels did not correlate with cytotoxicity and were high following SeV infection or poly(I:C) transfection but were undetectable following HSV-2 infection (Fig. 4L). To extend these observations *in vivo,* we measured IFNε by ELISA in uterine tissue homogenate and uterine lavage. IFNε levels were significantly higher (median: 3,935 pg/mL vs. 245.7 pg/mL, *P* = 0.0032 determined by Mann-Whitney *U* test) in the tissue compared to the lavage (Fig. 4M).

Together, these data indicate that IFNε is constitutively expressed and retained intracellularly within epithelial cells of the FRT, where it likely can signal after cellular damage or regular cell turnover at the surface of the epithelium.

### IFNε is basally expressed in and retained intracellularly in human intestinal organoids and mouse small intestine

**Fig 5:**
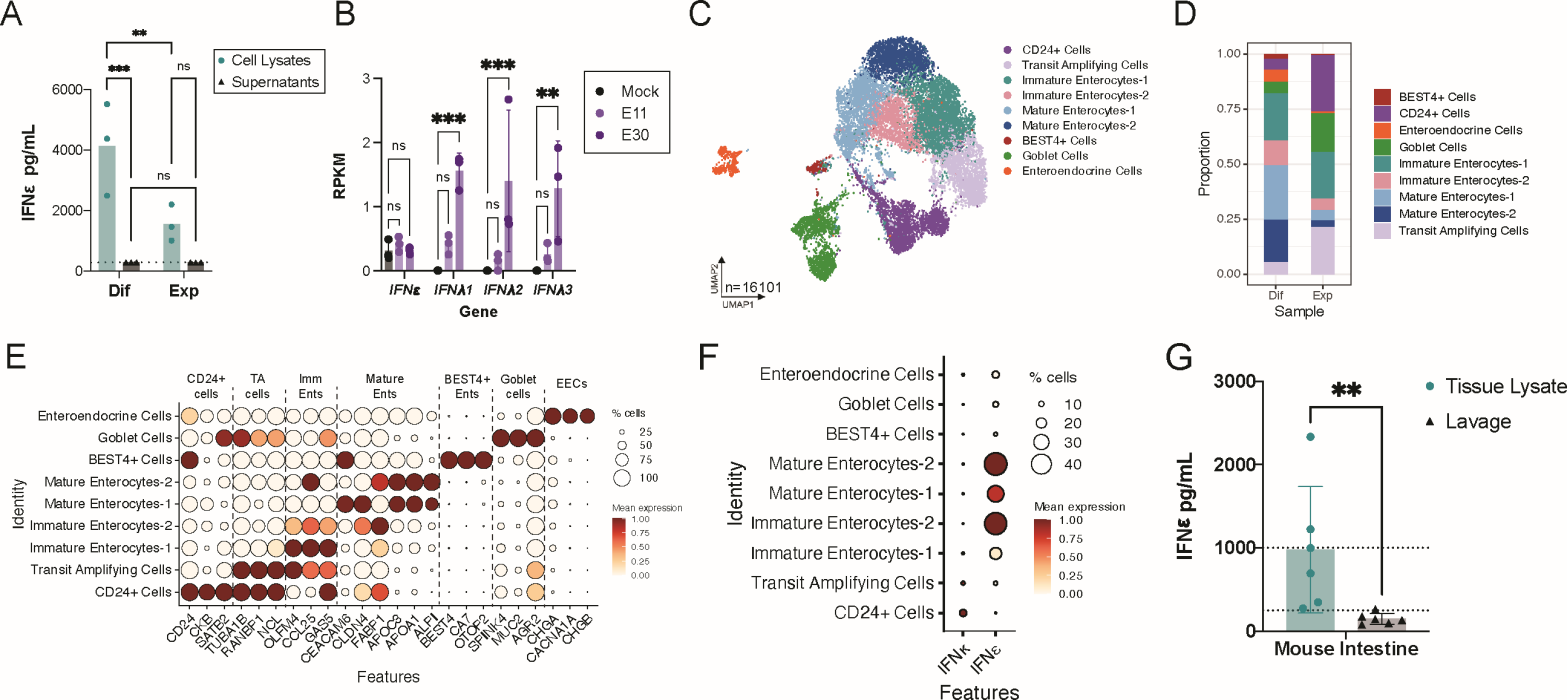
IFNε is expressed in human enterocytes, is retained intracellularly, and is not induced by infection. (**A–E**) Human enteroids were grown under expansion (Exp) or differentiating conditions (Dif) and then analyzed by scRNA-seq or bulk RNA-seq. (**A**) IFNε levels in supernatants and cell lysates from Exp and Dif enteroids, measured by ELISA. Data points represent IFNε concentration across experimental conditions, with significance determined by two-way ANOVA, followed by multiple comparisons to assess differences between groups (ns, not significant, **P < 0.01, ***P < 0.001). (**B**) Exp enteroids were infected with either echovirus 11 (E11) or echovirus 30 (E30) for 24 h. RPKMs for all interferons with detectable reads are represented. Significant differences were determined by Mann-Whitney *U* test (ns, not significant; **P < 0.01, ***P < 0.001). (**C**) UMAP plot depicting distinct cell clusters identified in the data set. Each cluster is represented by a unique color, indicating cellular heterogeneity and grouping based on transcriptomic profiles. (**D**) Bar plot depicting cell enrichment under Exp or Dif conditions. (**E**) Dot plot of canonical cell makers used to define clusters. Key and scale are shown on the right side. (**F**) *IFNε* and *IFNκ* expression in cell clusters. (**G**) IFNε protein levels were measured by ELISA from either small intestine tissue lysate or lavage from WT mice. Significant differences were determined by Mann-Whitney *U* test; **P < 0.01.

IFNε has been reported to be expressed in the gastrointestinal tract and may be associated with colitis (11). To determine if IFNε is retained intracellularly in the gastrointestinal tract as well as in the FRT, we examined cells and supernatants of human stem cell-derived enteroids. To include diverse GI cell types, human enteroids were grown in expansion media (Exp) to maintain a proliferative state with stem and progenitor cells or under differentiation conditions (Dif) to promote their maturation into specialized cells, including enterocytes, goblet cells, and Paneth cells (21, 22). Consistent with the FRT epithelium, IFNε was undetectable in the supernatants of both Exp and Dif enteroids and was only detected by ELISA in the cell lysates of both culture conditions (Fig. 5A). To determine if IFNε expression is influenced by infection, we infected Exp enteroids with two echoviruses, E11 and E30, and examined interferon expression broadly by RNA-seq. The only interferons with detectable reads were IFNλ and IFNε. *IFNλ1-3* were undetectable in mock samples, increased with E11 infection, and increased significantly with E30 infection. In comparison, IFNε was basally expressed and did not increase or vary significantly with either infection. (Fig. 5B). To define the basal expression of IFNε in the GI tract, we performed scRNA-seq of intestinal organoids under differentiation and expansion conditions. Enteroids cultured under these conditions contained CD24^+^ progenitor cells, transit amplifying cells, immature enterocytes, mature enterocytes, BEST4^+^ ionocytes, goblet cells, and enteroendocrine cells, which expressed canonical markers associated with these cell types (Fig. 5C through E). As expected, we found that Exp enteroids were enriched in CD24^+^ cells, transit amplifying cells, and immature enterocytes, and Dif enteroids were enriched in mature enterocytes and BEST4^+^ ionocytes and exhibited differences in cell type-specific expression (Fig. 5D and E; Fig. S6A and B). We found that *IFNε* was enriched in both mature enterocyte populations, as well as in a population of immature enterocytes (Fig. 5F; Fig. S6C). The only other IFN detectable in the data set was *IFNκ*, which was expressed at very low levels in CD24^+^ cells (Fig. 5F).

To extend these findings *in vivo,* we measured Ifnε levels by ELISA in both small intestine tissue lysate and lavage from WT mice. Ifnε was below detectable limits in lavage samples and was significantly more enriched in tissue lysate (mean: 845.7 pg/mL vs. 137.9 pg/mL, *P* = 0.0022, as determined by Mann-Whitney *U* test) (Fig. 5G). Altogether, these data indicate that IFNε is basally expressed in the small intestine in both humans and mice, primarily in enterocyte populations, and that it is retained intracellularly and not induced by infection in the intestine.

### IFNε is expressed in proximal, villus-tip enterocytes in the small intestine

**Fig 6:**
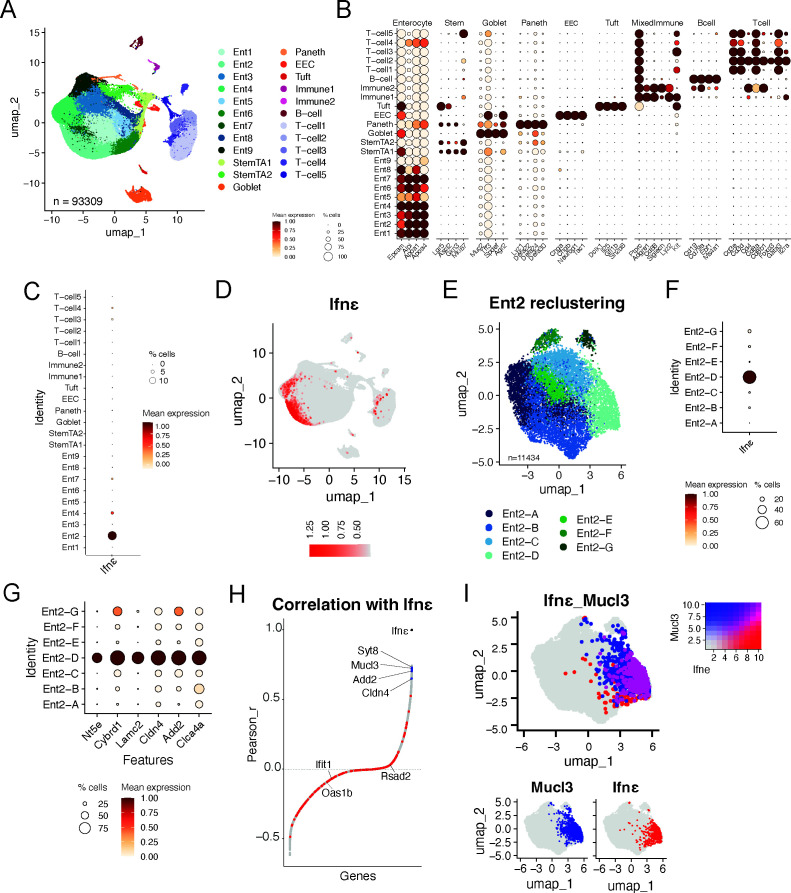
*Ifnε* is expressed in villous-tip enterocytes of the mouse small intestine. Single-cell suspensions were generated from WT and *Ifnε*^−/−^ mouse small intestines and analyzed by scRNA-seq. (**A**) UMAP plot revealed distinct cell clusters, including enterocytes (Ent), stem or transit-amplifying cells (Stem/TA), enteroendocrine cells (EEC), and immune cell populations expressing macrophage, dendritic cell, and mast cell markers. (**B**) Canonical marker genes used to define each cluster are shown in a dot plot. (**C and D**) *Ifnε* expression was detected primarily in enterocytes, as shown in both dot plots (**C**) and feature plots (**D**). (**E**) To further resolve enterocyte heterogeneity, cells within Ent2 were subsetted and reclustered, revealing distinct subclusters. (**F**) *Ifnε* expression across these subclusters is shown in a dot plot. (**G**) Dot plot shows the expression of top markers in Ent2-D, the cluster that expresses *Ifnε.* (**H**) Correlation analysis between *Ifnε* and all other genes expressed in Ent2 demonstrated no association with interferon-stimulated genes (ISGs, shown in red) but revealed strong correlations with villous tip-associated genes, including *Mucl3*. (**I**) A feature plot confirmed the overlap between *Ifnε* and *Mucl3* expression within Ent2, indicating that *Ifnε* is enriched in villous-tip enterocytes.

To further define the cellular expression patterns of *Ifnε* in the small intestine and assess its role in regulating ISG expression in the GI tract, we performed scRNA-seq on whole small intestines from WT and *Ifnε^−/−^* mice. All samples were merged and integrated together to correct for batch effects and to ensure the identification of cell types across experimental conditions, with 93,309 cells passing quality control. Clustering identified 23 unique clusters (Fig. 6A). All clusters were present across all samples, and there were similar proportions of each cluster in WT and *Ifnε^−/−^* samples (Fig. 6B; Fig. S7B). Clusters were annotated based on the expression of conserved marker genes, identifying major epithelial lineages (enterocytes, Paneth cells, goblet cells, tuft cells, enteroendocrine cells, and stem cells), as well as immune (T and B cells) and mesodermal populations (Fig. 6B). Consistent with our observations in enteroids, we observed that *Ifnε* expression *in vivo* was primarily restricted to enterocyte populations, with enterocyte cluster 2 (Ent2) exhibiting the highest proportion of *Ifnε*-positive cells (Fig. 6C and D). Because only ~10% of cells within Ent2 expressed *Ifnε*, we subsetted this cluster and performed reclustering to resolve enterocyte subpopulations and more precisely define the epithelial subset responsible for *Ifnε* expression (Fig. 6E). Reclustering resolved seven transcriptionally distinct enterocyte subpopulations, among which Ent2-D showed the highest *Ifnε* expression*,* with 60% of cells expressing the gene (Fig. 6F). Ent2-D was marked by the expression of duodenal cytochrome B (*Cybrd1*), a ferric reductase that is expressed in the brush border of duodenal enterocytes (23), as well as *Nt5e*, which is associated with villous tips (24) (Fig. 6G). These features indicate that *Ifnε* expression is enriched in proximal small intestinal enterocytes at the villous tips. To investigate this further, we calculated a villous index score for each cell type in the intestinal data set, defined as the ratio of villous tip-associated genes to the sum of villous tip- and villous bottom-associated genes, as described previously (24). The *Ifnε*-expressing subset of Ent2 displayed relatively high villous index scores, indicating enrichment in enterocytes positioned near the villous tips (Fig. S7C). This localization is consistent with previous observations that *Ifnε* is preferentially expressed in epithelial cells situated closest to the luminal surface of the colon (11). To assess whether *Ifnε*-expressing cells also exhibit elevated ISG levels, we performed a correlation analysis between *Ifnε* and all other genes within the subsetted Ent2 data set (Fig. 6H). No significant correlations were observed between *Ifnε* and ISGs, suggesting that *Ifnε* may serve functions in the small intestine distinct from basal antiviral gene induction. Instead, the top genes positively correlated with *Ifnε* included *Syt8*, *Cldn4*, *Add2*, and *Mucl3*, markers associated with villous tip enterocytes (Fig. 6H and I).

### Loss of *Ifnε* depletes inflammatory enterocyte subsets and increases susceptibility to enteric viral infection

**Fig 7:**
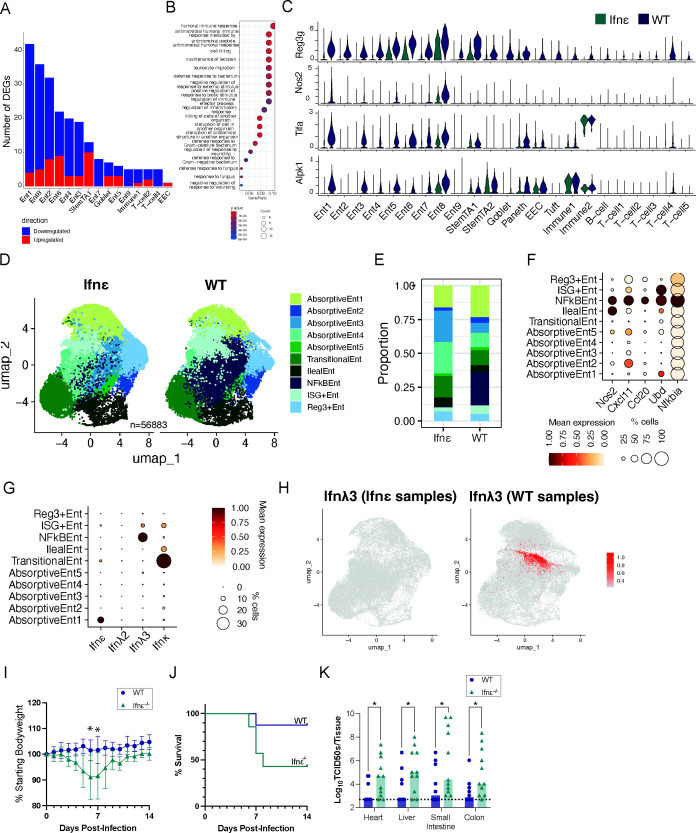
Loss of *Ifnε* depletes inflammatory enterocyte subsets and increases susceptibility to enteric viral infection. (**A**) Pseudo bulk analysis using DESEQ2 was performed for each cluster in the entire tissue intestinal single-cell RNA-seq data set introduced in Fig. 6. Total number of DEGs (in *Ifnε^−/−^* samples compared to WT) is shown for each cluster. Clusters not graphed (B-cell, Immune2, Paneth, STEMTA2, T-cell1, T-cell3, T-cell5, and Tuft) had no DEGs. (**B**) GO analysis was performed on the list of total identified DEGs, and pathways identified were graphed. Scales are shown below. (**C**) Violin plots of gene expression of DEGs identified in multiple clusters split by WT (dark blue) and *Ifnε^−/−^* (teal) samples. (**D–H**) All cells within enterocyte clusters 1–8 (enterocyte cluster 9 was excluded as its top markers were all mitochondrial genes) were subsetted from the original data set and reanalyzed. (**D**) Split UMAP depicting the clustering of the Ent 1–9 subclusters. (**E**) Bar plot shows the proportion of cells in each cluster in either WT or *Ifnε^−/−^* samples. (**F**) Dot plot shows the expression of the top markers of the NFkB enterocyte cluster. Key and scale are shown below. (**G**) Dot plot shows the expression of all interferons detected in the Ent 1–9 subcluster. (**H**) Feature plot shows the expression of *Ifnλ3* in either *Ifnε^−/−^* (left) or WT (right) samples. Scale is shown on the right side. (**I–K**) WT and *Ifnε^−/−^* mice were orally infected with 1 × 10^8^ PFU of CVB-H3 and weighed and monitored daily for signs of illness (**I and J**), or sacrificed at 4 days post-infection (dpi). Viral burdens in tissues (**K**) were determined by TCID50. Significant differences in weight (*P < 0.05) were calculated by *t*-test in GraphPad Prism. Differences in viral burden were calculated by Mann-Whitney *U* test (*P < 0.05).

To determine the broader impact of loss of *Ifnε* expression on intestinal homeostasis, we compared the small intestinal transcriptomes of WT and *Ifnε^−/−^* mice. Using pseudobulk differential expression analysis across all clusters in the data set, we identified genes differentially expressed between genotypes (Fig. 7A). DEGs were most abundant in enterocyte clusters, where they were enriched in pathways involved in antimicrobial and immune responses (Fig. 7B). Notably, genes such as *Reg3g* (antimicrobial lectin), *Nos2* (nitric oxide synthase), *Tifa* (NF-κB signaling adaptor), and *Alpk1* (innate immune kinase) were differentially expressed across multiple enterocyte subsets Fig. 7C; Fig. S8A and B). To further resolve enterocyte heterogeneity, we subsetted and reclustered Ent1-8, excluding Ent9, which was defined by mitochondrial gene expression and likely represented dying cells. This analysis revealed a distinct population of inflammatory enterocytes (NFκB^Ent^) present in WT intestines but largely absent from *Ifnε*^−/−^ samples (Fig. 7D and E). The NFκB^Ent^ cluster was characterized by the expression of *Nfkbia* (encoding an NFκB inhibitor), the NFκB regulator *Ubd*, and multiple inflammatory mediators, including *Nos2*, *Ccl20*, and *Cxcl11* (Fig. 7G). Although NFκB^Ent^ cells did not express *Ifnε*, they were the only enterocyte subset to express *Ifnλ3*, the gene encoding a member of the type III IFN family (Fig. 7G). *Ifnκ* was the only other IFN detected in enterocytes, but it was not expressed in NFκB^Ent^ cells. Strikingly, *Ifnλ3* expression was readily detected in WT enterocytes yet was completely absent in *Ifnε^−/−^* samples, suggesting that *Ifnε* may be involved in maintaining Ifnλ expression in the intestinal epithelium.

As Ifnλ is a central mediator of antiviral protection in the GI tract (25), we next tested whether *Ifnε*^−/−^ mice were more susceptible to enteric viral infection. To test this, we infected WT and *Ifnε^−/−^* mice with coxsackievirus B (CVB), an enterovirus that infects via the enteral route. Mice were infected with CVB3 (H3) by oral gavage. *Ifnε*^−/−^ mice exhibited significantly greater weight loss than WT controls (Fig. 7I) and showed increased mortality following infection (Fig. 7J). Notably, viral burdens in both the intestines and secondary organs, including the heart and liver, sites commonly affected by CVB, were markedly elevated in *Ifn*ε^−/−^ mice relative to WT controls (Fig. 7K). To better understand the mechanism by which Ifnε is protecting against disease in the intestine, we assessed WT and CVB-infected mice at 4 and 6 dpi for readouts of gross intestinal pathology, including colon length and gut leakiness measured by the presence of FITC-dextran in the serum following oral gavage. We observed no differences in colon length and were unable to detect any FITC-dextran in the serum of any of the conditions tested (data not shown). To further examine pathological differences, we submitted H&E-stained sections from intestinal Swiss rolls for pathological scoring and assessed immune infiltration and changes to the epithelium or mucosal architecture. The only observable changes were in inflammatory infiltrate and epithelial hyperplasia (Fig. S8F through H). At 4 dpi, all sections taken from *Ifnε^−/−^* had minimal or moderate inflammatory infiltrate in the mucosa of the intestine, while uninfected and WT samples had no infiltrate in most sections (Fig. S8F and G). While these results may indicate enhanced immune response or pathology in *Ifnε^−/−^* mice, these results did not extend to 6 dpi and would require additional testing to confirm.

To confirm that these differences were not due to microbiome alterations in WT or *Ifnε^−/−^* mice, which have been shown to impact CVB infection (26), we conducted 16S rRNA sequencing of the small ribosomal subunit from fecal samples of *Ifnε^+/−^* and *Ifnε^−/−^* littermates, thereby controlling for potential cage effects. The resulting +/− contained 2,695 OTUs, excluding those with a total count below three. On average, 76,503 quality-filtered reads were generated per sample, ensuring a robust analysis of microbiome composition (Fig. S9A). High-quality reads were classified using the Silva v. 138 reference database, and OTUs were aggregated at each taxonomic rank (Fig. S9B). Alpha diversity, measured by the Shannon index, was comparable between *Ifnε^−/−^* (mean = 4.004, SD = 0.015) and WT (mean = 3.865, SD = 0.262) mice (Fig. S9C). Differential abundance testing using DESeq2 revealed no OTUs with statistically significant differential abundance between WT and *Ifnε^−/−^* mice, indicating that microbiome composition was similar across genotypes.

Together, these findings indicate that *Ifnε* protects against enteric viral infection, thereby acting as a critical barrier that prevents systemic viral dissemination from the gastrointestinal tract.

## DISCUSSION

Antiviral defenses at mucosal surfaces are a crucial first-line defense against systemic infection. Type I IFNs play well-established roles in mucosal immunity and are induced by infection to elicit antiviral programs in target cells by signaling through the type I IFN receptor. Although IFNε is a type I IFN that also signals through the type I IFN receptor, it is not induced by infection or PAMPs. IFNε was initially characterized in the epithelium of the FRT, where it was proposed to be hormonally regulated, and more recently in the epithelium of the colon, indicating that IFNε has a role in immunity across many epithelial barriers. Here, we demonstrate that IFNε plays a constitutive, essential role in antiviral defenses across mucosal sites. Using an *Ifnε^−/−^* mouse model, we confirmed that IFNε provides innate antiviral protection within the FRT, where it is expressed in specific epithelial cell clusters in a location-dependent manner and is associated with basal ISG expression independent of the estrous cycle. Extending these findings to the intestine, we showed that IFNε is expressed in villous-tip enterocytes of the small intestine, where it supports inflammatory enterocyte subsets and sustains *Ifnλ3* expression. Loss of *Ifnε* led to depletion of these subsets, impaired *Ifnλ3* expression, and heightened susceptibility to enteric viral infection with systemic dissemination. Together, these results establish IFNε as a critical component of mucosal immunity, providing sustained antiviral defense across both reproductive and gastrointestinal epithelial tissues.

Using an existing single-cell atlas of normally cycling mice (19), we found that *Ifnε* is expressed in distinct epithelial cell types in each tissue of the FRT: luminal and secretory cells in the uterus, columnar cells in the vagina, ciliated cells in the oviducts, and superficial cells in the cervix. In each epithelial population that expressed *Ifnε,* we examined its expression levels at each estrous cycle stage. While the *Ifnε* expression showed some fluctuations with the estrous stage within individual tissues, it was consistently observed at all stages, with no patterns of fluctuation that were consistent across all tissues. The cell types that express *Ifnε* in the uterus—secretory and luminal epithelial cells—make up the layers of the endometrium that expand in preparation for embryo implantation during estrous (27), which could result in the higher gross *Ifnε* transcripts at a tissue level that others have observed in the uterus. These findings refine our understanding of *Ifnε* expression in the FRT, revealing its presence in distinct epithelial populations across tissues and estrous stages, suggesting a stable role in mucosal immunity.

Previous studies have identified a role for IFNε in modulating inflammation in colitis models and shown Ifnε expression by IHC in the colon (11). To further define the cell types that express IFNε in the intestine, we analyzed *IFNε* expression in human enteroids and in mouse intestinal scRNA-seq data sets and observed *Ifnε* primarily in enterocytes, as well as in select T-cell populations. Murine enterocytes enriched for *Ifnε* also expressed markers of villus-tip localization as well as a duodenal marker, indicating that *Ifnε* expression is enriched in proximal villous-tip enterocytes. Unlike in the FRT, where Ifnε expression correlated with ISG enrichment, intestinal analyses revealed no such association. Instead, *Ifnε*^−/−^ mice showed loss of key antimicrobial genes, including *Nos2* and *Reg3g*, across multiple enterocyte clusters. Strikingly, ~25% of enterocytes in WT mice formed an immune-activated cluster characterized by the expression of chemokines and *Ifnλ3,* which was nearly absent in *Ifnε^−/−^* mice. Prior studies have shown that tonic IFNλ signaling establishes a basal antiviral state that restricts enteric pathogens (28) and identified pDCs as a source of tonic Ifnλ (29). While we were unable to capture pDCs in our single-cell data sets in any significant amounts, our findings suggest select enterocytes can also produce tonic Ifnλ. Since tonic Ifnλ is induced by microbiota, microbial differences between mice may account for differences in cell types that express Ifnλ. Our data show that IFNε functions as an upstream regulator of tonic Ifnλ in enterocytes, maintaining baseline antiviral readiness in the intestinal epithelium. In accordance with this, *Ifnε^−/−^* mice exhibited increased weight loss and mortality and higher viral titers in the intestines, heart, and liver after oral infection with the enteric virus coxsackivirus B (CVB). Taken together, these data define a role for IFNε throughout the intestines, where it is expressed in enterocytes and controls viral infection.

We initially expected that IFNε-expressing cell types would share common features; however, we observed distinct anatomical differences among the epithelial populations expressing *IFNε*. IFNε has been identified in tissues by IHC and is often found in the epithelial cells lining the lumen of tissues (1, 5, 11, 16). This pattern suggests that IFNε expression may be influenced by specific environmental or spatial triggers within these barriers. Recently, the transcription factor ELF3 was identified as a driver of *Ifnε* expression in the mouse uterus (8), where it appears to support tissue-specific immune readiness. ELF3 is an epithelium-specific transcription factor involved in regulating the expression of genes essential for barrier function and epithelial integrity. While we consistently observed *Elf3* expression almost exclusively in *Ifnε*-expressing clusters throughout the FRT, in the intestine, *Elf3* was detectable in nearly all cell types, indicating that there are other factors that control *IFNε* expression. This finding indicates that distinct transcriptional programs may regulate IFNε expression across different tissues, potentially adapting its expression to localized immune needs and environmental cues. Together, these observations suggest that IFNε expression is not only anatomically specialized but may also be regulated by diverse transcriptional drivers in response to the unique environmental contexts of each barrier tissue.

In both our scRNA-seq data set of the mouse intestine and of the uterus, we observed small differences in immune cell clusters in *Ifnε^−/−^* mice. In the intestine, a small portion (1%–2%) of two T-cell clusters expressed *Ifnε,* and several immune cell clusters, including T cells, had differentially expressed genes in our *Ifnε^−/−^* mice that included cytokines such as *Il17a*. In the uterus, both macrophages and DCs had a handful of differentially expressed genes. It remains to be determined if these differences in immune cells contribute to the phenotypes we see in *Ifnε^−/−^* mice, but these observations are in line with other groups that have reported that immune cells respond to recombinant IFNε treatment by becoming activated, expanding, or upregulating ISGs (11, 30, 31). It has been observed that Ifnε knockout mice have reduced NK cell numbers in the uterus, a phenotype that results in part from decreases in IL-15 signaling in *Ifnε^−/−^* macrophages/monocytes (10). In mouse models of colitis, *Ifnε*^−/−^ mice exhibit decreased levels of T_regs_ in the intestines, which exacerbate intestinal pathology (1, 11). These findings indicate that IFNε likely has a role in regulating resident tissue immune cells as well as those that respond during infection.

Although many cell types respond to IFNε after exogenous treatment with recombinant protein, the cellular targets of IFNε signaling have not been well-defined in models where it is basally expressed. In our scRNA-seq analyses of the FRT, we observed ISG expression restricted to cells that also basally expressed IFNε. This ISG expression is dependent on IFNε, as *Ifnε*^–/–^ mice lack ISG expression in the absence of IFNε signaling, suggesting an autocrine role for IFNε. Moreover, in primary human vaginal and uterine epithelial cell lines, we found that IFNε was detectable by ELISA in cell lysates but was undetectable in cell supernatants unless the cells were cultured under lytic conditions. We observed similar results in human enteroids, where IFNε expression could be readily detected by scRNA-seq and ELISAs of cellular lysates, but not in supernatants, indicating that IFNε is retained intracellularly. Another study also reported that IFNε is retained intracellularly *in vitro* due to inefficient processing through the Golgi. Instead, IFNε accumulated in the cytoplasm and did not exert antiviral activity (20). Retaining IFNε intracellularly within specific epithelial layers vulnerable to infection may prevent aberrant immune activation by a constitutive interferon while still providing basal antiviral protection. In cases of cellular damage or viral infection, IFNε could also function as a damage-associated molecular pattern (DAMP), where it may be released into the extracellular space and act in a paracrine manner to signal neighboring mucosal cells or immune cells. In mucosal tissues with frequent epithelial cell turnover, such as the intestine or uterine endometrium, IFNε could be released from cells during the normal process of epithelial turnover. Future studies will be crucial in elucidating the precise mechanisms by which IFNε initiates signaling and identifying the specific cellular contexts where its autocrine activity plays a protective role, which may provide new insights into its unique function at mucosal barriers.

Together, our findings underscore the unique role of IFNε in maintaining mucosal immunity as a constitutively expressed IFN within epithelial barriers. By providing baseline antiviral protection while remaining poised for activation upon cellular damage or infection, IFNε serves as both a steady immune defender and a potential DAMP, capable of alerting neighboring cells to pathogenic threats. The enrichment of IFNε in villous-tip enterocytes highlights the importance of spatial positioning within the intestinal epithelium, placing this IFN at an interface where viral entry is most likely to occur. Moreover, the dependence of *Ifnλ3* expression on IFNε reveals a previously unappreciated regulatory axis between constitutive and inducible IFNs that shapes epithelial antiviral programs. This dual functionality helps prevent unwanted immune activation that could lead to autoimmune pathology, while ensuring rapid and localized antiviral responses when needed. Future studies aimed at clarifying the intracellular signaling mechanisms of IFNε and its DAMP-like functions may reveal new therapeutic approaches for enhancing mucosal immunity without triggering systemic inflammation.

## MATERIALS AND METHODS

### Mice

IFNε knockout (*Ifnε^−/−^*) mice were generated by iGONAD in-house (detailed below) and then bred in-house. WT mice were ordered from the Jackson Laboratory (#000664) and then bred in-house.

### Generation of IFNε knockout mice using iGONAD

IFNε knockout (*Ifnε^−/−^*) mice were generated using the iGONAD technique, as previously described (17, 18). Briefly, female C57BL/6J mice were mated with C57BL/6J males. On the morning of day E0.7, CRISPR/Cas9-mediated gene editing was performed on pregnant mice by injecting a ribonucleoprotein (RNP) complex consisting of Cas9 protein and two guide RNA (gRNAs) targeting exon 1 (AACGGATTCCCTTCCAATTG[TGG] and AAGTCTTAGCTGCGCTTCAC[CGG], where the PAM sites are indicated with [PAM]) of the *Ifnε* gene into the oviducts. The two gRNAs were designed to flank and induce double-strand breaks, leading to the deletion of a 263-base-pair fragment within exon 1, which resulted in a frameshift mutation and knockout of IFNε expression. After injection, the oviducts were electroporated to facilitate the delivery of the ribonucleoprotein (RNP) complex into presumed one-cell stage zygotes. After gene editing surgery, embryos continued to develop, and pregnant females were allowed to deliver naturally. Genotyping of the resulting pups was carried out by extracting genomic DNA from toe biopsies using a lysis buffer containing 50 mM Tris-base (pH 8.0), 50 mM KCl, 2.5 mM EDTA, 0.45% IGEPAL CA-630 (NP40), 0.45% Tween-20, and 20 mg/mL proteinase K, followed by incubation at 94°C for 10 min. PCR amplification was performed with primers flanking the gRNA target sites (forward: TCCCAGAACTGGAGTGGT; reverse: AAGAGCCAACAGGGGATTT) using TITANIUM Taq DNA Polymerase (Takara). The knockout was further confirmed by Sanger sequencing using a primer upstream of the gRNA sites (TCCCAGAACTGGAGTGGT) to verify successful deletion of the *Ifnε* gene. Ifnε^−/−^ mice were backcrossed to C57BL/6J for at least six generations before use in experiments to establish a stable knockout line. Once established, Transnetyx developed a custom qPCR-based genotyping assay to streamline the identification of *Ifnε* knockout alleles in subsequent generations.

### Viruses

HSV-2 (strain 333) was provided by Helen Lazear (University of North Carolina at Chapel Hill) and propagated and titrated using a plaque assay in Vero cells, which were cultured in Dulbecco’s modified Eagle medium (DMEM) supplemented with 5% fetal bovine serum (FBS). CVB3 (H3 strain), echovirus 11 (strain Gregory), and echovirus 30 (strain Bastianni) were propagated and titrated in HeLa cells cultured in modified Eagle’s medium (MEM) supplemented with 5% FBS. For titration, serial dilutions of virus were added to Vero (HSV-2) or HeLa (CVB, E11, E30) cell monolayers and incubated for 1 day at room temperature. Following binding, cells were overlaid with a mixture of phenol red-free MEM and 0.5% agarose and incubated for 48–72 h at 37°C and 5% CO_2_. The overlay was removed after incubation, and plaques were subsequently visualized by staining with crystal violet. Sendai virus (Cantell strain) was purchased from Charles River (Pl-1, SV).

### HSV-2 infections in mice

Eight- to ten-week-old virgin female mice were used for all experiments. For HSV-2 infections, females were treated with 2 mg of medroxyprogesterone acetate (MPA, NDC 66993-270-83) by subcutaneous injection 5 days prior to infection to synchronize females in diestrus. Diestrus was confirmed by vaginal cytology as previously described (32); 100 or 10,000 PFU of HSV-2 was given by vaginal inoculation in a 10 µL volume. HSV-2 was diluted in sterile PBS. Mice were weighed and scored for pathology daily according to the following criteria: 0, no inflammation; 1, vaginal edema and redness; 2, hair loss or ulceration localized to the vaginal-anal area; 3, severe ulceration, extensive hair loss extending beyond the vaginal-anal area; 4, overt disease signs; 5, paralysis; and 6, death. Vaginal washes were taken by pipetting 110 µL of PBS into and out of the vagina five times. Vaginal lavages were titrated by plaque assay on Vero cells as described above.

### CVB-H3 infections in mice

All CVB-H3 infections were performed in 8- to 10-week-old male mice. Mice were infected by oral gavage with 1 × 10^8^ pfu in 100 µL of PBS. For viral burden experiments, mice were sacrificed after 4 days, and tissues were harvested and homogenized in DMEM using an Omni International Bead Ruptor Elite (19-042E) at 4 m/s for 1 min using 2.8-mm ceramic beads in 2 mL tubes (Omni International 19-628). Viral burdens were quantified using a TCID₅₀ assay on HeLa cells. Serial 10-fold dilutions of virus samples were prepared in complete medium and added to confluent monolayers of HeLa 7B cells in 96-well plates. Cells were incubated for 3 days (37°C, 5% CO₂). Cells were fixed and stained with crystal violet to visualize cytopathic effect (CPE). The dilution at which 50% of wells showed CPE was recorded as the TCID₅₀ endpoint, using the Reed-Muench method to calculate the viral titer.

### Histopathology and RNA scope

Intestines were harvested and Swiss-rolled using a published protocol (33), fixed overnight in 10% NBF, and sent to Histowiz for paraffin embedding, sectioning, and H&E staining. Six intestinal sections from each time point and genotype tested were submitted for blind pathological scoring at Histowiz. Sections were assessed for inflammatory infiltrate, epithelial cell changes (hyperplasia, goblet cell loss, cryptitis, crypt abscess, and erosion), and changes to mucosal architecture (ulceration, tissue granulation, irregular crypts, crypt loss, and villous blunting). RNA scope was performed using the RNA Scope Multiplex Fluorescent Reagent Kit v2 (ACD #323100) according to the manufacturer’s instructions and Ifnε probes (ACD 816081-C2).

### Generation of uterine single-cell suspension for scRNA-seq

Naturally cycling WT and Ifnε mice in estrus were confirmed by vaginal cytology (32). The whole uterus was dissected and placed in 1× DPBS on ice until all tissues were harvested. Uteruses were minced into 1–2 mm pieces with dissecting scissors and placed into 2.5 mL of D-PBS supplemented with Ca and Mg (Gibco, 14040117). Once all tissues were minced, 2.5 mL of pre-warmed 2× dissociation medium was added; 2× dissociation medium was made by dissolving 4 mg/mL of collagenase A (Roche, 10103578001) and 2 U/mL of DNase I (Roche, 04536282001) in dispase (Corning, 354235). Samples were incubated for 30 min at 37 degrees and were shaken vigorously for 30 s every 5 min. After incubation, enzymes were inactivated by adding 0.5 mL of FBS, and samples were placed on ice. Samples were pushed through a 100-µm filter (VWR 732-2759) using the plunger of a 1 mL syringe. Cells were pelleted at 400 × *g* for 3 min at 4°C and resuspended in 1 mL of ice-cold PBS; 2 mL of RBC lysis buffer (Invitrogen, 00-4333-57) was added for 1 min with a single inversion to mix, and then, 10 mL of 1% FBS-DPBS was added to each sample. Cells were pelleted at 400 × *g* for 3 min at 4°C and resuspended in 500 µL of ice-cold 1% FBS-PBS. Cells were counted and resuspended at a final volume of 1.5 × 10^6^ cells/mL in 1% FBS-DPBS. Cells were kept on ice and immediately utilized for scRNA-seq processing.

### Generation of intestinal single-cell suspension for scRNA-seq

The entire length of the small intestine was dissected from age-matched, 8-week-old WT and Ifnε male mice and processed into a single cell suspension as previously described (34). Briefly, intestinal contents were flushed with 1× HBSS (Sigma H4641), opened longitudinally, and minced into 1 mL pieces and then washed twice with 1× HBSS. Tissues were digested for 20 min in digestion media: 1× HBSS, 5% FBS (Gibco A56707-01), 5 mM EDTA (RPI E14000-250), and 1 mM DTT (Sigma D0632). Samples were shaken vigorously to help release epithelial cells, and then, the supernatant containing epithelial cells was passed through a 100 µm cell strainer after allowing the residual tissue segments to settle at the bottom of the tube. Samples were spun down at 2,000 RPM for 5 min, resuspended in 1 mL of room temperature PBS, and then 2 mL of RBC lysis buffer (Invitrogen, 00-4333-57) was added and mixed into the sample by inverting. RBC lysis was performed for 1 min at room temperature; 10 mL of ice-cold 5% FBS-HBSS was added to each sample, and then, the samples were strained through a 40 µM filter. Cells were counted and resuspended at a final volume of 1.5 × 10^6^ cells/mL in 5% FBS-HBSS. Cells were kept on ice and immediately utilized for scRNA-seq processing.

### Enteroid growth and single-cell preparation

We used previously established human enteroids (35, 36). Human enteroid culture and differentiation were based on the published protocols for adult stem cells (22, 37, 38). For expansion, enteroids were passaged by single-cell dissociation in TrypLE (Gibco) at 37°C for 5 min, resuspended in 40 µL Matrigel (Corning) domes, and overlayed with ExM for 7 days. For differentiation, enteroids were mechanically disrupted in DPBS, resuspended in 40 µL Matrigel domes, grown in patterning medium (PaM) for 16 days, and in differentiation medium (DiM) for 3 days. For single-cell analysis, enteroids were expanded (Exp) or differentiated (Dif), scraped into cold DPBS, pelleted for 5 min at 400 × *g*, resuspended in TrypLE, and incubated at 37°C for 15 min. Enteroids were mechanically dissociated by pipetting up and down 30 times, diluted in basal media, pelleted for 5 min at 400 × *g*, resuspended in DPBS with 1% BSA, and filtered through a 30-µm strainer. Basal media contained 10 mM HEPES, 2 mM L-glutamine, and 100 U/mL penicillin-streptomycin in Advanced DMEM/F12 (Gibco). ExM additionally contained 1× B27 (Invitrogen), 1 mM N-acetylcysteine (Sigma), 50 ng/mL mouse EGF (Peprotech), 100 ng/mL mouse Wnt3a, 500 ng/mL mouse R-spondin, 100 ng/mL human noggin (R&D Systems), 1× N2 (Invitrogen), 10 mM nicotinamide (Sigma), 100 ng/mL human IGF-1, 50 ng/mL human FGF-2 (Peprotech), 500 nM A83-01 (Tocris), 10 nM PGE2 (R&D Systems), and 10 nM (leu15)-gastrin I (Millipore). Patterning media (PaM) additionally contained 1× B27, 1 mM N-acetylcysteine (NAC), 50 ng/mL mouse EGF, 100 ng/mL mouse Wnt3a, 500 ng/mL mouse R-spondin, 100 ng/mL human noggin, 500 nM A83-01, and 2 ng/mL IL-22 (Peprotech). Differentiation media (DiM) additionally contained 1× B27, 1 mM N-acetylcysteine (NAC), 50 ng/mL mouse EGF, 50 ng/mL BMP-2, and 50 ng/mL BMP-4 (Peprotech).

### Enteroid infection with echoviruses

Expansion enteroids were infected for 24 h with either echovirus 11 (E11) strain Gregory or echovirus 30 (E30) strain Bastianni. Each dome/well of enteroids was incubated for 1 h with 400 µL of inoculum at a concentration of 1 × 10^6^ PFU/mL. After 1 h, the inoculum was removed and replaced with fresh media. RNA was collected after 24 h and sent for bulk RNA sequencing.

### Single-cell RNA-seq library preparation and data analysis

Publicly available data sets from cycling mouse tissue (19) were downloaded from ArrayExpress (E-MTAB-11491), and data sets from human intestine were downloaded from GEO (accession numbers: GSE185224, GSE171620, and GSE125970) (39–41).

For uterus tissue from WT and *Ifnε^−/−^* mice, single-cell RNA-seq libraries were prepared from ~10,000 cells/mouse using the 10× Genomics Chromium Single Cell 3' Reagent Kit (v2 chemistry, Manual Part #CG00052) following the manufacturer’s protocol. Briefly, single-cell suspensions were loaded into the 10× Chromium Controller to capture and barcode individual cells. Libraries were constructed, and sequencing was performed on an Illumina NovaSeq 6000 system (Illumina, San Diego) using an S2 flow cell, providing an average sequencing depth of ~61,000 reads per cell. After sequencing, raw base call (BCL) files were processed with the 10× CellRanger pipeline (v6.1.2, 10× Genomics) for demultiplexing, alignment to the mouse reference genome (GRCm38), and quantification of gene expression. Quality control and post-processing were carried out using the CellRanger count and aggregate functions to generate gene expression matrices. Data analysis was performed using the Seurat package (42, 43) (v4.0) in R. Cells were filtered to include those with at least 700 but no more than 12,000 unique expressed genes. Additionally, cells with over 10% mitochondrial gene content were excluded from further analysis. To correct for batch effects, data sets from individual animals were normalized using the SCTransform() function (v2) in Seurat, and integration was performed using the FindIntegrationAnchors() and IntegrateData() functions. Dimensionality reduction was conducted via principal component analysis (PCA) using the RunPCA() function. Cell clusters were identified using Louvain clustering with the FindClusters() function in Seurat. The optimal clustering resolution was determined by evaluating clustering stability across a range of resolutions (0.2 to 1.0) using the clustree() package, with a resolution of 0.3 selected for downstream analysis. For combined analyses of wild-type (WT) and *Ifnε^−/−^* data sets, previously integrated data sets were merged and further integrated using the Harmony algorithm (44) (v1.0) for batch correction and alignment. Differential expression analysis between clusters was performed using the Wilcoxon rank sum test implemented in Seurat’s FindAllMarkers() function. Genes with a log_2_ fold change > 0.25 and an FDR-adjusted *P*-value < 0.05 were considered significantly differentially expressed. Marker genes for each cluster were identified by searching for positively enriched genes using the aforementioned criteria.

Human fetal enteroid single-cell RNA-seq libraries were similarly prepared, sequenced, and analyzed with minor differences; ~10,000 cells/sample were targeted, providing an average sequencing depth of ~61,000 reads per cell. After sequencing, BCL files were processed with the 10× CellRanger pipeline (v7.0.1, 10× Genomics) for demultiplexing, alignment to the human reference genome (GRCh38), and quantification of gene expression. Cells were filtered to include those with at least 3,000 but no more than 10,000 uniquely expressed genes. Cells with less than 2% or more than 20% mitochondrial gene content were excluded from further analysis. Individual samples were merged and integrated using the FindIntegrationAnchors() and IntegrateData() functions into Exp and Dif. Percent mitochondrial genes, feature count, read count, and the difference in G2M and S phase scores were regressed. For combined analysis, Exp and Dif were merged and further integrated using the Harmony algorithm (44) (v1.0) for batch correction and alignment.

For intestine tissue from WT and *Ifnε^−/−^* mice, libraries were prepared targeting 20,000 cells/mouse using 10x Genomics v4 GEM-x 3′ chemistry. Libraries were sequenced on a NovaSeq X plus using two lanes of a 25B flow cell. Data were analyzed similarly to the uterine tissue, but with some key differences. Data were analyzed using Seurat version 5.3.0 (45). Cells were filtered to include those with at least 1,000 but no more than 12,000 unique genes. Cells with greater than 15% mitochondrial gene counts were excluded from further analysis. Individual samples were merged into a split object and normalized and scaled using NormalizeData() and ScaleData() with *Ifn*ε explicitly retained as a variable feature. WT and *Ifnε^−/−^* samples were integrated separately using Harmony Integration and the IntegrateLayers() function. Unique clusters were identified using a resolution of 0.6. To improve the detection of lowly expressed transcripts, we applied the adaptively thresholded low-rank approximation (ALRA) algorithm (46). Differentially expressed genes between WT and *Ifnε^−/−^* clusters were determined using DESeq2.

### Primary cell PAMP treatments and infections

Primary human cells were purchased from Lifeline Cell Technologies (FC-0078, FC-0083) and thawed into 24-well or 6-well plates according to the manufacturer’s instructions. At 90%–100% confluency, cells were treated with PAMPS or infected at the following concentrations: polyIC float (InvivoGen, tlrl-picw) 10 µg/mL; polyIC transfection (InvivoGen, tlrl-picw) 1 µg/mL (0.5 µg/well); cGAMP transfection (InvivoGen, tlrl-nacga23-02) 10 µg/mL; SeV Cantell 20 HAU or 200 HAU per well; HSV-2 333 MOI of 1 or 10; and LPS float (InvivoGen, tlrl-eklps) 100 ng/mL. cGAMP and polyIC were transfected using xTREMEgene9 (Roche, XTG9-RO) according to the manufacturer’s instructions. Supernatants or cell lysates were collected at 20–24 h, depending on the experiment, and used for ELISA, qRT-PCR, bulk RNA sequencing, or LDH assay (details below).

### qPCR and bulk RNA sequencing

RNA was extracted from cells using the Qiagen RNeasy mini kit (74104) according to the manufacturer’s instructions. *IFNε*, *IFNβ*, and *ACTB* RNA levels were detected by qRT-PCR using IDT PrimeTime Std qPCR Assay Primer Probes: *IFNε* Hs.PT.58.4812867; *IFNβ1* Hs.PT.58.39481063.g, ACTB: Hs.PT.39a.22214847, and Itaq one-step kit (BIO-RAD 172-5140). qRT-PCR was run using a Bio-Rad Opus. For bulk RNA sequencing, libraries were prepared by the Duke Center for Computational Biology and Genomics (GCB) using a mRNA stranded KAPA hyperprep kit. Sequencing was performed on an Illumina Novaseq X Plus using 100 bp paired-end sequencing and targeting 10 billion reads across all samples. Reads were aligned to the human genome using Qiagen CLC Genomics (v20). Differential expression analysis was performed using DESeq2. Briefly, raw count data were imported into R, and DESeq2 (47) was used to normalize counts and identify differentially expressed genes between specified conditions. Genes with an adjusted *P*-value < 0.05 and log_2_ ±2-fold change were considered significantly differentially expressed.

### ELISA and LDH activity assay

Human IFNε and human IFNβ protein levels were detected by ELISA (R&D Systems, DY9667-05, R&D Systems DY814-05). IFNε and IFNβ levels were normalized to total protein quantified by Pierce BCA Assay (23227). Cellular lysates were generated using M-PER Mammalian Protein Extraction Reagent (Thermo Scientific, 78501). Lactate dehydrogenase activity was quantified using an LDH-Blue Cytotoxicity Assay (InvivoGen rep-ldh-1).

### IFNε quantification in mouse uterine and intestinal lavage and lysate

Age-matched, 8-week-old, virgin female mice were treated with 100 μg of Depo-Estradiol by intraperitoneal injection 1 day prior to harvest to synchronize mice in estrous. Mice were euthanized, and we harvested the uterus and first 10 cm of intestine and lavaged with either 200 μL (uterus) or 500 μL (intestine) with PBS. Tissue was homogenized in M-PER Mammalian Protein Extraction Reagent (Thermo Scientific, 78501). IFNε levels in lavage and tissue homogenate were measured by ELISA Biomatik (EKC37135). IFNε levels in tissues were consistently higher than the upper limit of detection and were extrapolated from the standard curve.

### 16S ribosomal sequencing

Fecal samples were collected from adult *Ifnε^+/−^* and *Ifnε^−/−^* littermates and sent to Microbiome Insights for 16S sequencing and analysis. Specimens were placed into a MoBio PowerMag Soil DNA Isolation Bead Plate. DNA was extracted following MoBio’s instructions on a KingFisher robot. Bacterial 16S rRNA genes were PCR-amplified with dual-barcoded primers targeting the V4 region (515F 5′-GTGCCAGCMGCCGCGGTAA-3′, and 806R 5′-GGACTACHVGGGTWTCTAAT-3′), as previously described (48). Amplicons were sequenced with an Illumina MiSeq using the 300-bp paired-end kit (v.3). Sequences were denoised, taxonomically classified using Silva (v. 138) as the reference database, and clustered into 97%-similarity operational taxonomic units (OTUs) with the mothur software package (v. 1.44.1) (49), following the recommended procedure (https://www.mothur.org/wiki/MiSeq_SOP; accessed November 2020). Alpha diversity was estimated with the Shannon index on raw OTU abundance tables after filtering out contaminants. The significance of diversity differences was tested with ANOVA or a linear mixed model, depending on the study design. To estimate beta diversity across samples, we excluded OTUs occurring with a count of less than 3 in at least 5% of the samples and then computed Bray-Curtis indices. We visualized beta diversity, emphasizing differences across samples, using principal coordinate analysis (PCoA) ordination. Variation in community structure was assessed with permutational multivariate analyses of variance (PERMANOVA) with treatment group as the main fixed factor and using 999 permutations for significance testing. A dot-dash circle in an ordination plot represents the center of each cluster (50). All analyses were conducted in the R environment.

## Data Availability

Fastq files were uploaded to SRA under the BioProject ID PRJNA1329228 (mouse uterus and intestine), PRJNA1336277 (enteroids), or PRJNA1403047 (infected enteroids).

## SUPPLEMENTAL MATERIAL

Supplemental Figures mbio.00340-26-s0001.pdf Figures S1 to S9.

ASM does not own the copyrights to Supplemental Material that may be linked to, or accessed through, an article. The authors have granted ASM a non-exclusive, world-wide license to publish the Supplemental Material files. Please contact the corresponding author directly for reuse.
