# Supplementary material for: Constitutive interferon epsilon expression shapes antiviral epithelial states in the female reproductive tract and intestine
Source: mBio. 2026 May 5;17(6):e00340-26. doi: 10.1128/mbio.00340-26 (PMC13251463; doi:10.1128/mbio.00340-26)
Supplement: Supplemental Figures — Figures S1 to S9. [file mbio.00340-26-s0001.pdf]

A

$Ifn\varepsilon^{-/-}$	ATGGTTCACAGACAGCTCCCTGAAACGGTGTTGCTGCTCTTGGTTTCTTCCACTATCTTC
WT	ATGGTTCACAGACAGCTCCCTGAAACGGTGTTGCTGCTCTTGGTTTCTTCCACTATCTTC
$Ifn\varepsilon^{-/-}$	TCCCTAGAACCGAAACGG--
WT	TCCCTAGAACCGAAACGGA**AACGGATTCCCTTCCAATTG**TGGATGAACAGAGAAAGCCTA
$Ifn\varepsilon^{-/-}$	---
WT	CAACTACTGAAACCTTTGCCAAGCTCGTCAGTCCAGCAGTGTCTAGCACACAGGAAGAAT
$Ifn\varepsilon^{-/-}$	--
WT	TTCCTGCTTCCTCAGCAGCCTGTGAGTCCTCACCAGTACCAAGAGGGACAGGTGCTGGCT
$Ifn\varepsilon^{-/-}$	--
WT	GTTGTGCACGAGATCCTTCAGCAGATCTTCACGCTCCTCCAGACACATGGGACTATGGGC
$Ifn\varepsilon^{-/-}$	---CGGCAGCTGGAATAC
WT	ATTTGGGAGGAAAACCATATAGAAA**AAGTCTTAGCTGCGCTTCAC**CGGCAGCTGGAATAC

Supplemental Figure 1. (A) Sequence alignment following Sanger sequencing of genomic DNA isolated from an $Ifn\varepsilon^{-/-}$ mouse (top) or wild-type (WT, bottom) mouse. Bold red sequences denote location of gRNAs (gRNA1 and gRNA2). Dashes denote sequence missing in the $Ifn\varepsilon^{-/-}$ mouse.

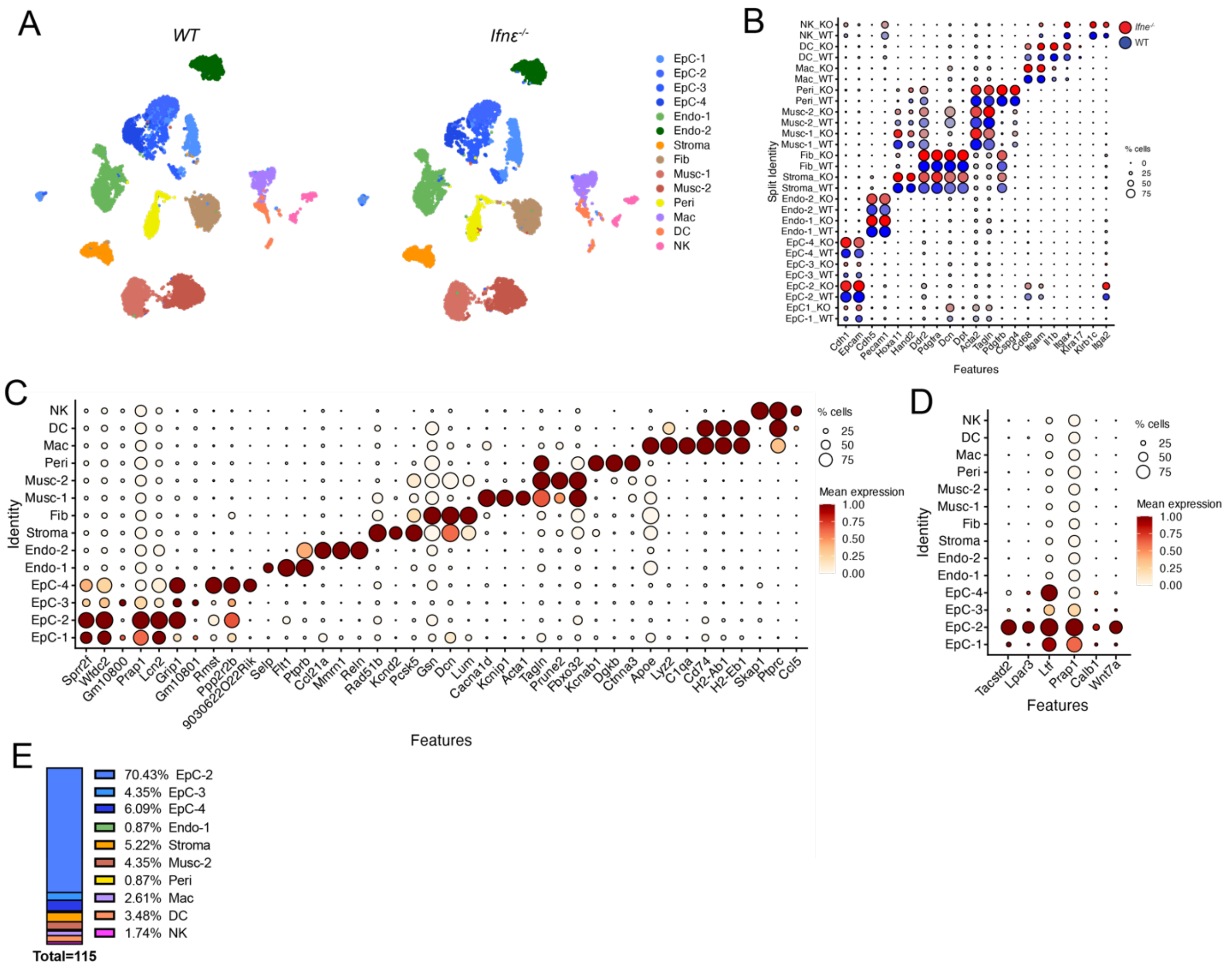

Supplemental Figure 2. (A), UMAP of cell clusters in uterine tissue isolated from WT (left) of Ifnε$^{-/-}$ mice (right) split by genotype. (B), DotPlot of canonical cell markers in WT (blue) or *Ifnε*$^{-/-}$ (red) mice. **(C),** DotPlort of cell type markers in the combined dataset from WT and *Ifnε*$^{-/-}$ mice. Scale and key at right. (D), DotPlot of luminal cell markers in WT and *Ifnε*$^{-/-}$ dataset. Key and scale at right. (E), Bar plot indicating the cell clusters associated with differentially upregulated genes (115 total) in WT mice compared to *Ifnε*$^{-/-}$ mice. The percent is shown at right.

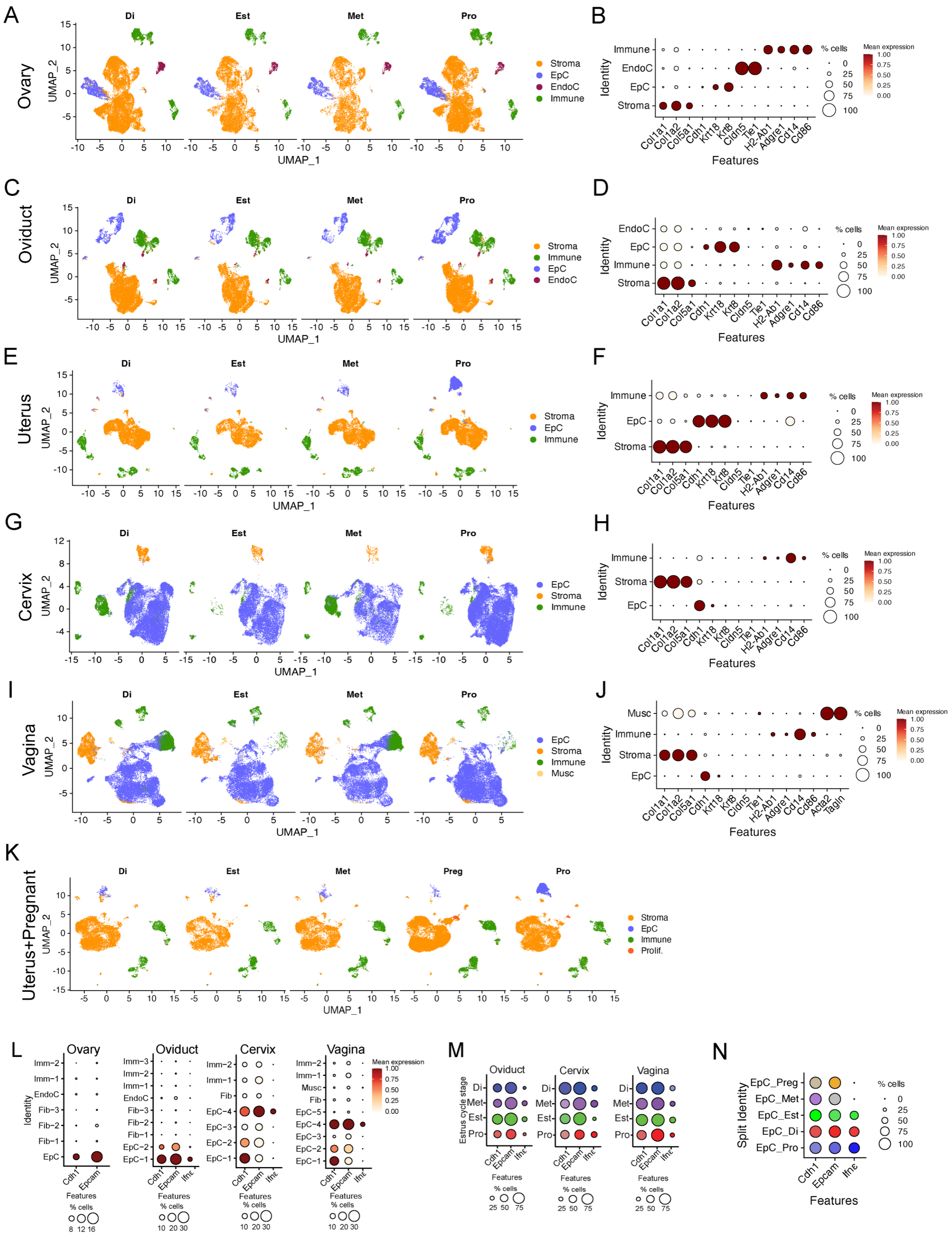
A
Ovary
Di
Est
Met
Pro
UMAP_2
UMAP_1
Stroma
EpC
EndoC
Immune
B
Identity
Features
% cells
Mean expression
Col1a1
Col1a2
Col5a1
Cdh1
Krt18
Krt8
Cldn5
Tie1
H2-Ab1
Adgre1
Cd14
Cd86
C
Oviduct
D
E
Uterus
F
G
Cervix
H
I
Vagina
Musc
J
Acta2
Tagln
K
Uterus+Pregnant
Preg
Prolif.
L
Imm−2
Imm−1
Imm−3
Fib−3
Fib−2
Fib−1
Fib
EpC−1
EpC−2
EpC−3
EpC−4
EpC−5
Epcam
Ifnε
M
Estrus cycle stage
N
Split Identity
EpC_Preg
EpC_Met
EpC_Est
EpC_Di
EpC_Pro

Supplemental Figure 3. (A, C, E, G, I) UMAPs showing clustering of stromal (Stroma, orange), epithelial (EpC, blue), immune (Immune, green), endothelial (EndoC, red), and muscular (Musc, yellow) cells within distinct phases of the estrous cycle the different tissues of the female reproductive tract (FRT) shown at left. **(D, F, H, J),** DotPlot of canonical markers in Oviduct (D), Uterus (F), Cervix (H), Vagina (J), and Uterus (J). **(K),** UMAP of uterine tissue throughout the estrous cycle and pregnant uterus (Preg). **(L)** Dotplots showing expression of cadherin-1 (*Cdh1*), *Epcam*, and *Ifnε* in each cell cluster of the ovary, oviduct, cervix, and vagina. There was no detectable *Ifnε* in the ovary. **(M-N)** DotPlots of cadherin-1 (*Cdh1*), *Epcam*, and *Ifnε* in epithelial cells of the oviduct, cervix, vagina (M) or uterus (N) split by phase of the estrous cycle (M) or estorus cycle and pregnancy (in orange, N).

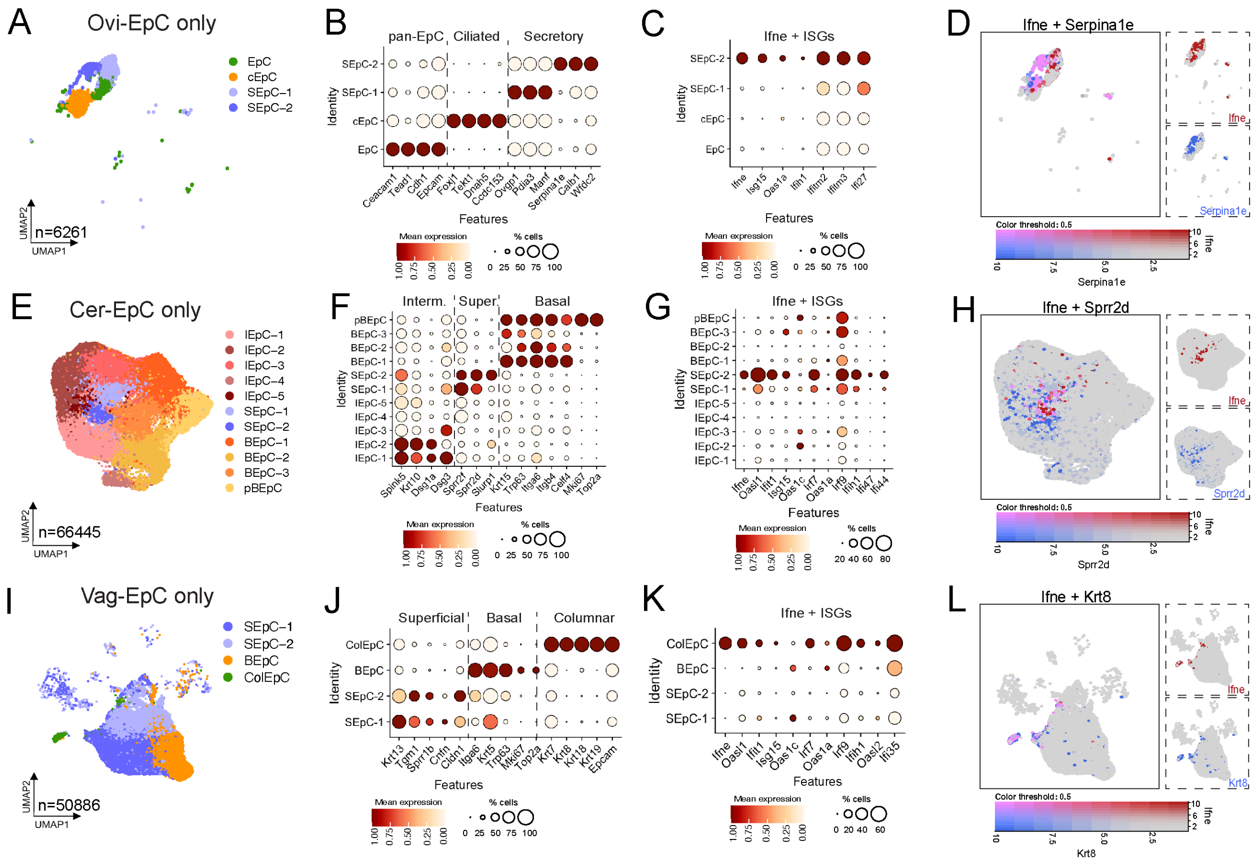

Supplemental Figure 4. Epithelial cell clusters from Figure 2 were reclustered. **A,E,I)** UMAPS showing clustering of epithelial cell types within each tissue. Oviduct **(A)**: general epithelial cells (Epc), ciliated epithelial cells (cEpC), and secretory epithelial cells (SEpC-2). Cervix **(E)**: intermediate epithelial cells (IEpC), superficial epithelial cells (SEpC), basal epithelial cells (BEpC), and parabasal epithelial cells (pBEpC). Vagina **(I)**: superficial epithelial cells (SEpC), basal epithelial cells (BEpC), and Columnar epithelial cells (ColEpC). **B,F,J).** Dot plots showing markers of each epithelial subpopulation. **C,G,K)** Dot plot showing Ifnε and ISG expression within each cluster. **D,H,L)** Feature plots showing expression and coexpression of Ifnε and a top marker for the cluster in which it is expressed.

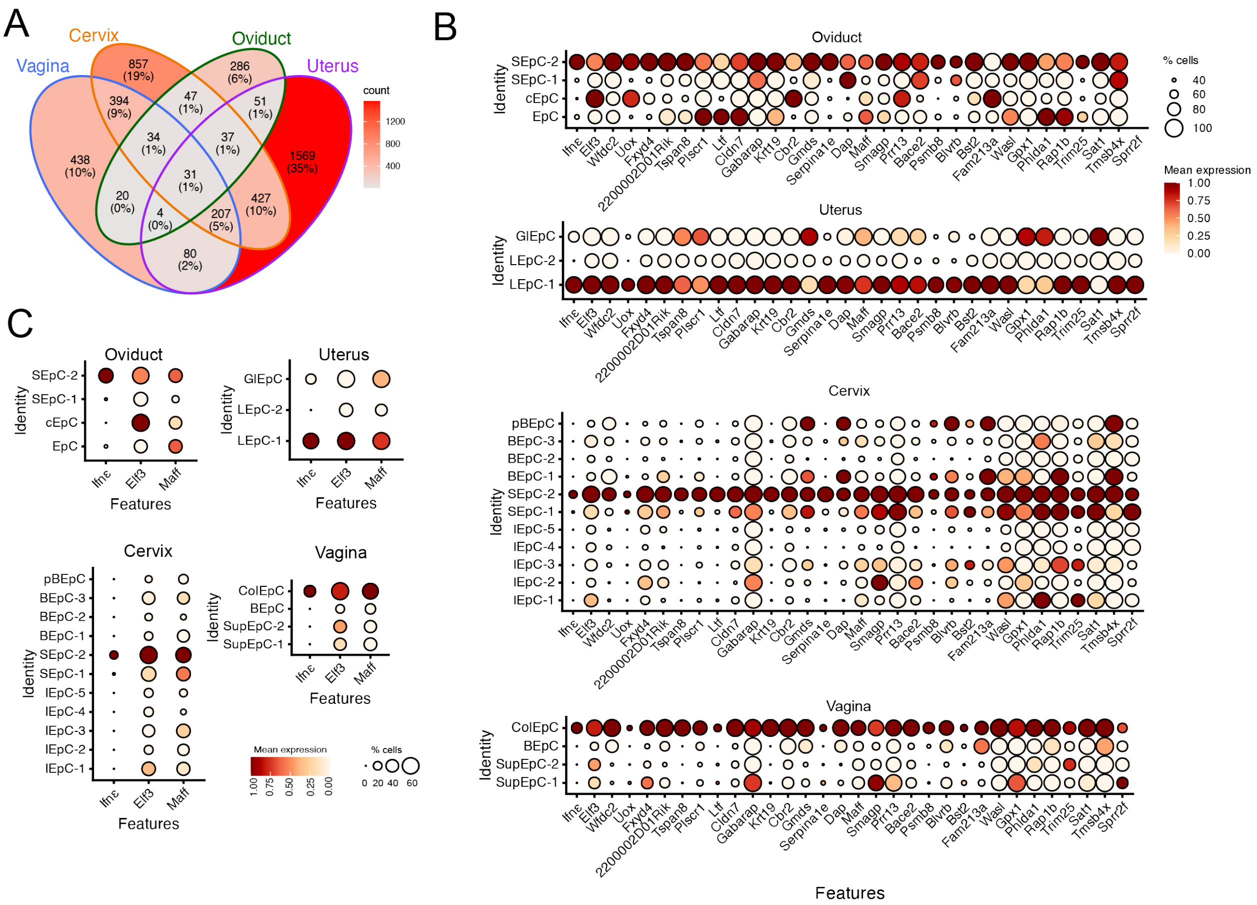

Supplemental Figure 5. (A), Venn diagram denoting the overlap of gene enriched in Ifnε-expressing clusters from Oviduct (green), Uterus (purple), Cervix (orange), and Vagina (blue). Key at right with red indicating the highest number of genes. The percent of overlap is also shown. **(B),** DotPlot of the genes differentially enriched in *Ifnε*-expressing clusters in Oviduct (top), Uterus (second from top), Cervix (second from bottom), and Vagina (bottom). Key and scale at right. **(C),** DotPlot of the expression of *Ifnε*, *Elf3*, and *Maff* in Oviduct, Uterus, Cervix, or Vagina as indicated. Key and scale at bottom.

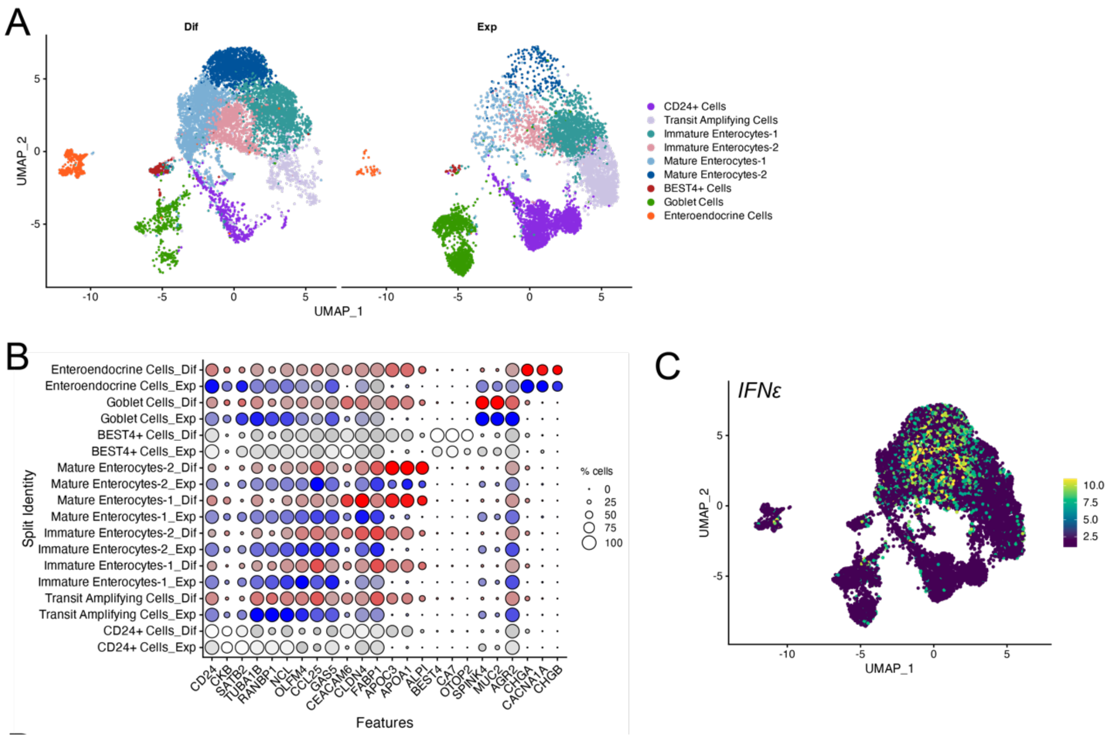

Supplemental Figure 6. (A), UMAP of cell clusters in enteroids cultured under expansion (Exp) or differentiated (Dif) conditions. **(B),** DotPlot of canonical makers in enteroids split by either Dif (red) or Exp (blue) growth conditions. **(C),** FeaturePlot of *IFNE* in enteroids. Scale at right.

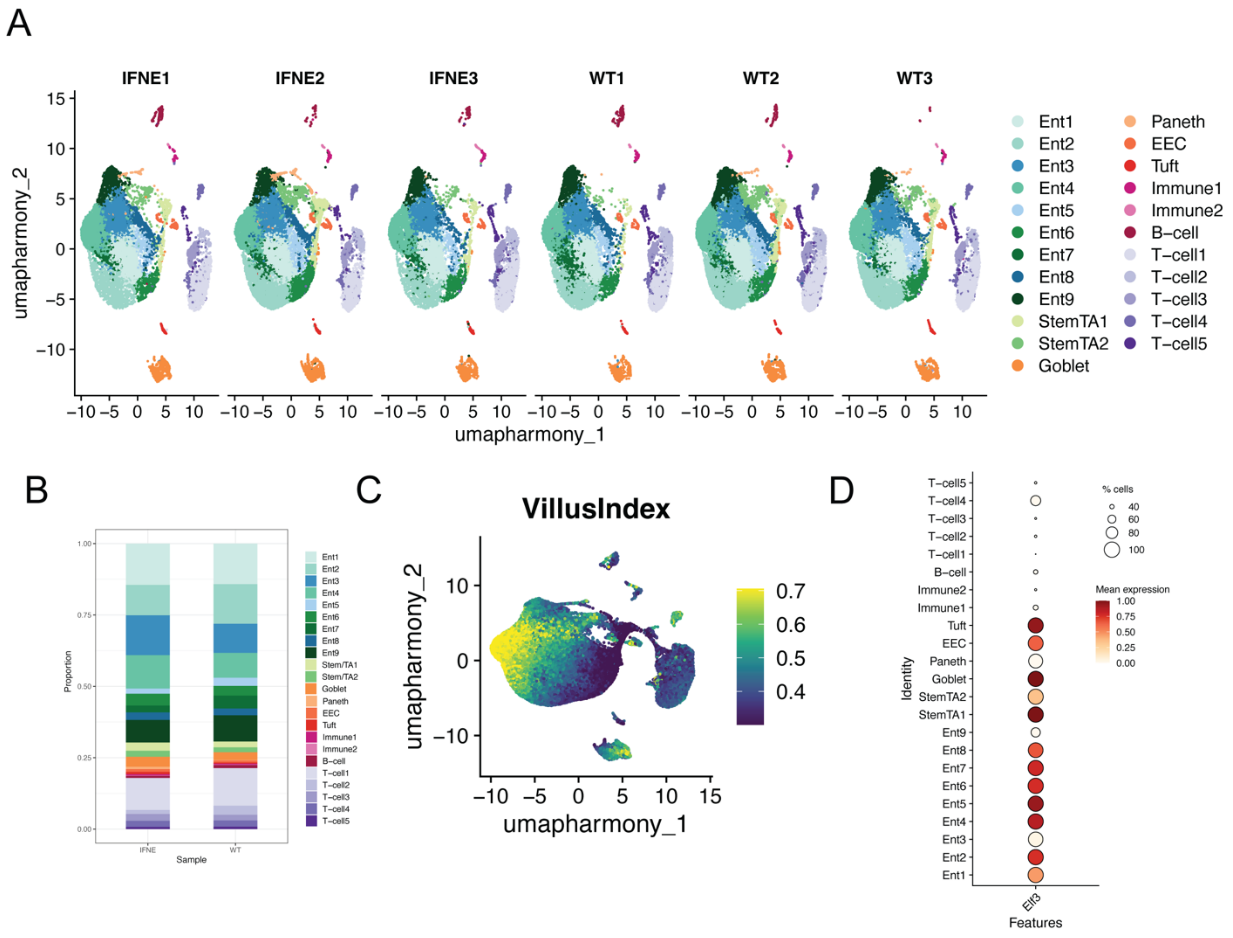

Supplemental Figure 7: (A), UMAP of cell clusters in intestinal tissue isolated from WT or Ifnε$^{-/-}$ mice (right) split by sample. **(B)** Bar plot showing the proportion of cells in each cluster split by WT or Ifnε$^{-/-}$ mice **(C)** Feature plot showing a villus index score for each cell as calculated as follows: (expression top genes/(expression top genes + expression bottom genes). 62 bottom landmark genes and 43 top landmark genes were included that were identified in Moor et al. 2018. **(D)** Dotplot showing proportion and expression levels of *Elf3* in each cluster. Scale and key to the right.

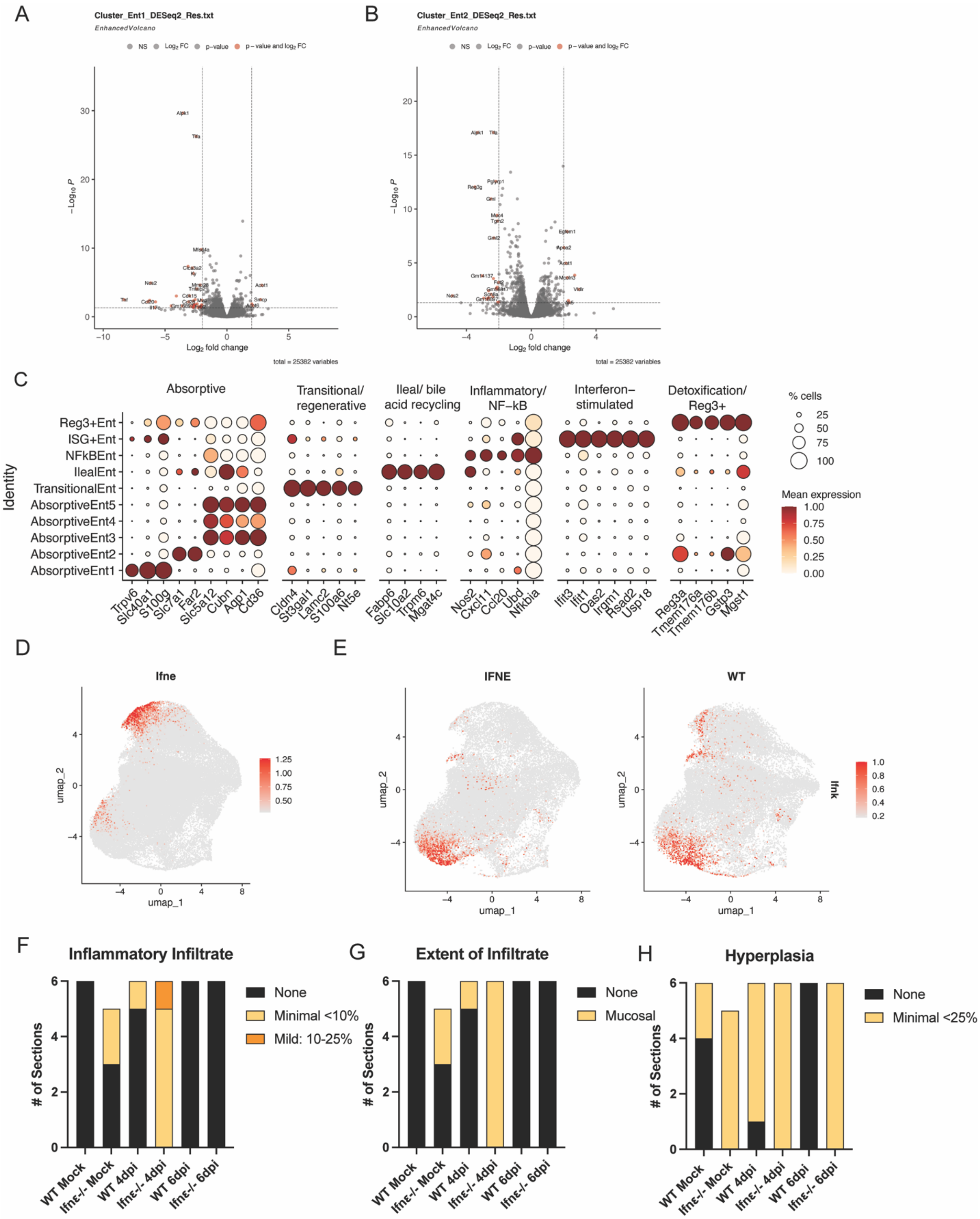

Supplemental Figure 8: (A-B) Volcano plots showing genes differentially expressed in Ifnε$^{-/-}$ samples compared to WT in clusters Ent1 (A) and Ent2 (B). DEGs were calculated by DESeq2. **(C).** DotPlot showing markers used to define types of enterocyte clusters. Key and scale to the right. **(D)** FeaturePlot showing Ifne expression. Scale to the right. **(E)** FeaturePlot showing Ifnk expression split by Ifnε$^{-/-}$ and WT samples. Scale to the right. **(F-H)** Ifnε$^{-/-}$ and WT mice were

infected orally with CVB-H3 and harvested at 4 or 6 days post-infection. Intestines were dissected, swiss-rolled, paraffin-embedded and sectioned. H&E stained sections were scored for pathological scoring. All abnormal results, including inflammatory infiltrate and epithelial hyperplasia are included here.

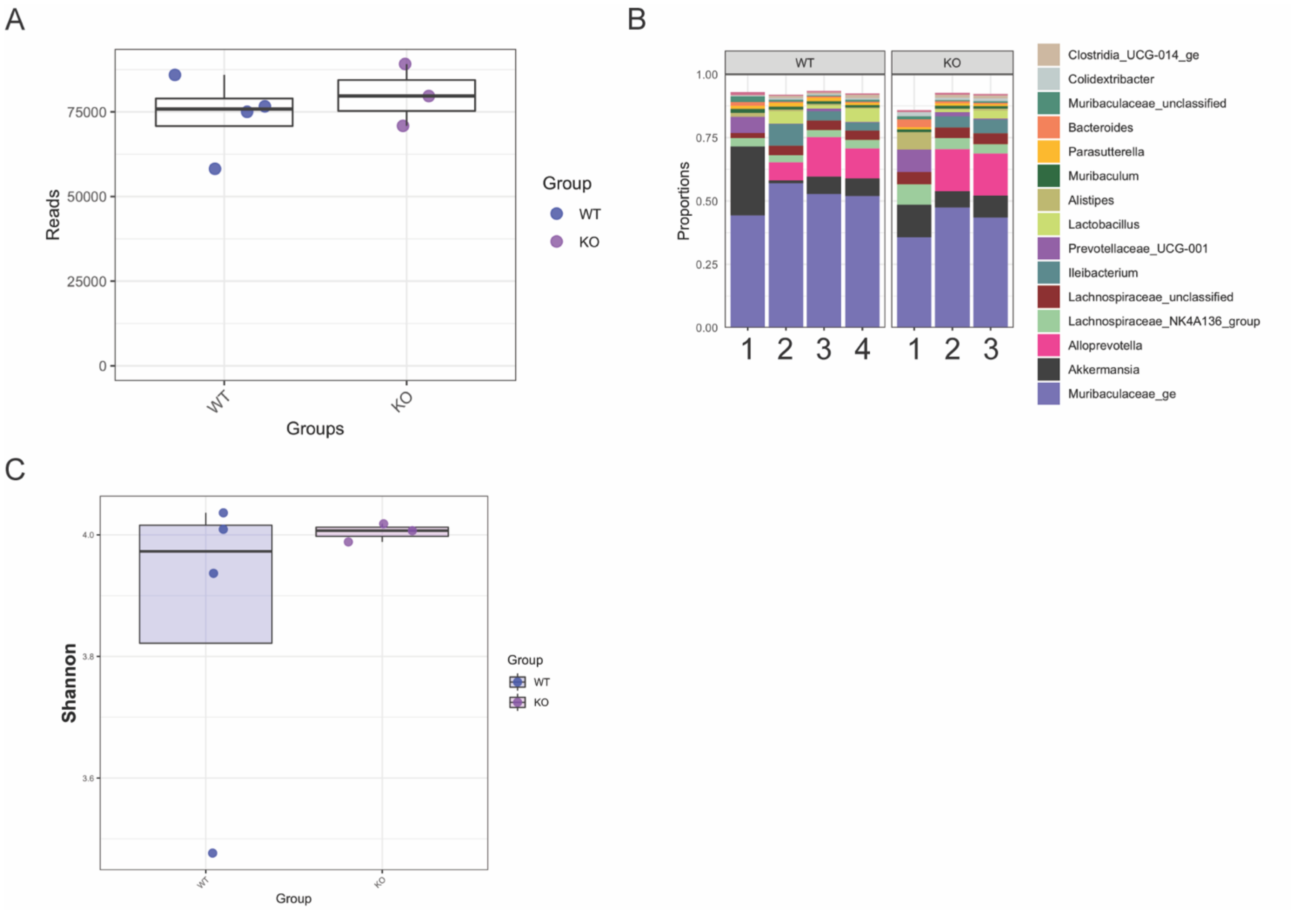

Supplemental Figure 9. (A), Total reads generated from 16S sequencing on fecal samples from *$Ifn^{+/-}$* (WT) and *$Ifn\varepsilon^{-/-}$* (KO) littermates **(B),** Proportions of OTUs at each taxonomic rank in each sample **(C)** Alpha diversity measured by Shannon Index for *$Ifn\varepsilon^{+/-}$* (WT) and *$Ifn\varepsilon^{-/-}$* (KO) littermates.
